# Establishment of a novel human CIC-DUX4 sarcoma cell line, Kitra-SRS, with autocrine IGF-1R activation and metastatic potential to the lungs

**DOI:** 10.1038/s41598-019-52143-3

**Published:** 2019-11-01

**Authors:** Sho Nakai, Shutaro Yamada, Hidetatsu Outani, Takaaki Nakai, Naohiro Yasuda, Hirokazu Mae, Yoshinori Imura, Toru Wakamatsu, Hironari Tamiya, Takaaki Tanaka, Kenichiro Hamada, Akiyoshi Tani, Akira Myoui, Nobuhito Araki, Takafumi Ueda, Hideki Yoshikawa, Satoshi Takenaka, Norifumi Naka

**Affiliations:** 1 Department of Orthopaedic Surgery, Osaka University Graduate School of Medicine, 2-2 Yamadaoka, Suita, Osaka 565-0871 Japan; 2 Department of Orthopaedic Surgery, Yao Municipal Hospital, 1-3-1 Ryugecho, Yao, Osaka 581-0069 Japan; 3 Department of Orthopaedic Surgery, Kawachi General Hospital, 1-31 Yokomakura, Higashiosaka, Osaka 578-0954 Japan; 4 Musculoskeletal Oncology Service, Osaka International Cancer Institute, 3-1-69 Otemae, Chuo-ku, Osaka 541-8567 Japan; 5 Compound Library Screening Center, Osaka University Graduate School of Pharmaceutical Sciences, 1-6 Yamadaoka, Suita, Osaka 565-0871 Japan; 6 Department of Orthopaedic Surgery, Ashiya Municipal Hospital, 39-1 Asahigaokacho, Ashiya, Hyogo 659-8502 Japan; 7 Department of Orthopaedic Surgery, Osaka National Hospital, 2-1-14 Hoenzaka, Chuo-ku, Osaka 540-0006 Japan

**Keywords:** Cancer models, Sarcoma, Targeted therapies, Metastasis, Growth factor signalling

## Abstract

Approximately 60–70% of *EWSR1*-negative small blue round cell sarcomas harbour a rearrangement of *CIC*, most commonly *CIC-DUX4*. *CIC-DUX4* sarcoma (CDS) is an aggressive and often fatal high-grade sarcoma appearing predominantly in children and young adults. Although cell lines and their xenograft models are essential tools for basic research and development of antitumour drugs, few cell lines currently exist for CDS. We successfully established a novel human CDS cell line designated Kitra-SRS and developed orthotopic tumour xenografts in nude mice. The *CIC-DUX4* fusion gene in Kitra-SRS cells was generated by t(12;19) complex chromosomal rearrangements with an insertion of a chromosome segment including a *DUX4* pseudogene component. Kitra-SRS xenografts were histologically similar to the original tumour and exhibited metastatic potential to the lungs. Kitra-SRS cells displayed autocrine activation of the insulin-like growth factor 1 (IGF-1)/IGF-1 receptor (IGF-1R) pathway. Accordingly, treatment with the IGF-1R inhibitor, linsitinib, attenuated Kitra-SRS cell growth and IGF-1-induced activation of IGF-1R/AKT signalling both *in vitro* and *in vivo*. Furthermore, upon screening 1134 FDA-approved drugs, the responses of Kitra-SRS cells to anticancer drugs appeared to reflect those of the primary tumour. Our model will be a useful modality for investigating the molecular pathology and therapy of CDS.

## Introduction

*CIC* rearrangement is the genetic abnormality that is generally detected in approximately 60–70% of *EWSR1*-negative small blue round cell sarcomas^1,2^. This rearrangement most commonly connects *CIC* (19q13) to *DUX4* (4q35 or 10q26); some tumours harbour *CIC* rearrangements with non-*DUX4* partner genes, including *FOXO4*, *LEUTX*, *NUTM1*, and *NUTM2A*^1,3–8^. Although *CIC*-rearranged sarcomas are conventionally referred to as “Ewing-like sarcomas”, recent studies have described a significant distinction between *CIC*-rearranged sarcomas and *EWSR1*-rearranged Ewing sarcomas^1,2,9^. *CIC-DUX4* sarcoma (CDS) occurs predominantly in children and young adults, and usually arises in the somatic soft tissues with only rare osseous involvement^1,2,10–12^. Because patients with CDS show an aggressive clinical course with a high metastatic rate and quickly develop resistance to chemotherapy, the median survival is less than 2 years, an inferior overall survival compared with Ewing sarcoma patients^2,13,14^. An effective therapy for CDS remains to be established, and novel therapeutic strategies are urgently required.

The *CIC-DUX4* fusion gene is implicated in oncogenesis, tumour development, and metastatic capability^7,15^. *CIC*, a human homologue of *capicua* in *Drosophila*, is a member of the high-mobility group box superfamily of transcription factors that normally inhibits *ETV1/4/5* expression and regulates receptor tyrosine kinase (RTK) signalling pathways^16–18^. *DUX4* is a double-homeobox gene that belongs to the family of double homeodomain transcriptional activators and is located within the D4Z4 sequence, which is a 3.3-kb tandem repeat located at the subtelomeric region of 4q35 or 10q26^19^. The *CIC-DUX4* fusion oncoprotein remarkably potentiates the transcriptional activity of *CIC* and activates the expression of downstream targets, including *ETV1/4/5*, which is a member of the E26 transformation-specific (ETS) family of transcription factors^7,9,15^. However, the details of the molecular pathogenesis of CDS and therapeutic targets for this sarcoma are not fully understood.

Although cell lines and xenograft models are useful tools for preclinical research and understanding of the mechanism of drug sensitivity, because CDS patients are rare, few cell lines have been established^7,20^; furthermore, no CDS cell lines are available for both *in vitro* and *in vivo* studies. In our current study, we first established and characterized a novel human CDS cell line termed Kitra-SRS, and then developed orthotopic tumour xenografts with metastatic potential to the lungs in nude mice. Kitra-SRS cells exhibited autocrine activation of the insulin-like growth factor 1 (IGF-1)/IGF-1 receptor (IGF-1R) pathway, and the IGF-1R selective inhibitor, linsitinib, suppressed Kitra-SRS cell growth *in vitro* and *in vivo*. In addition, by 1134 FDA-approved drug screening, ten anticancer drugs exhibited remarkable antiproliferative effects against Kitra-SRS cells, and the responses of Kitra-SRS cells to anticancer drugs appeared to be reflective of those of the primary tumour.

## Results

### Clinical background and tumour histology

**Figure 1:**
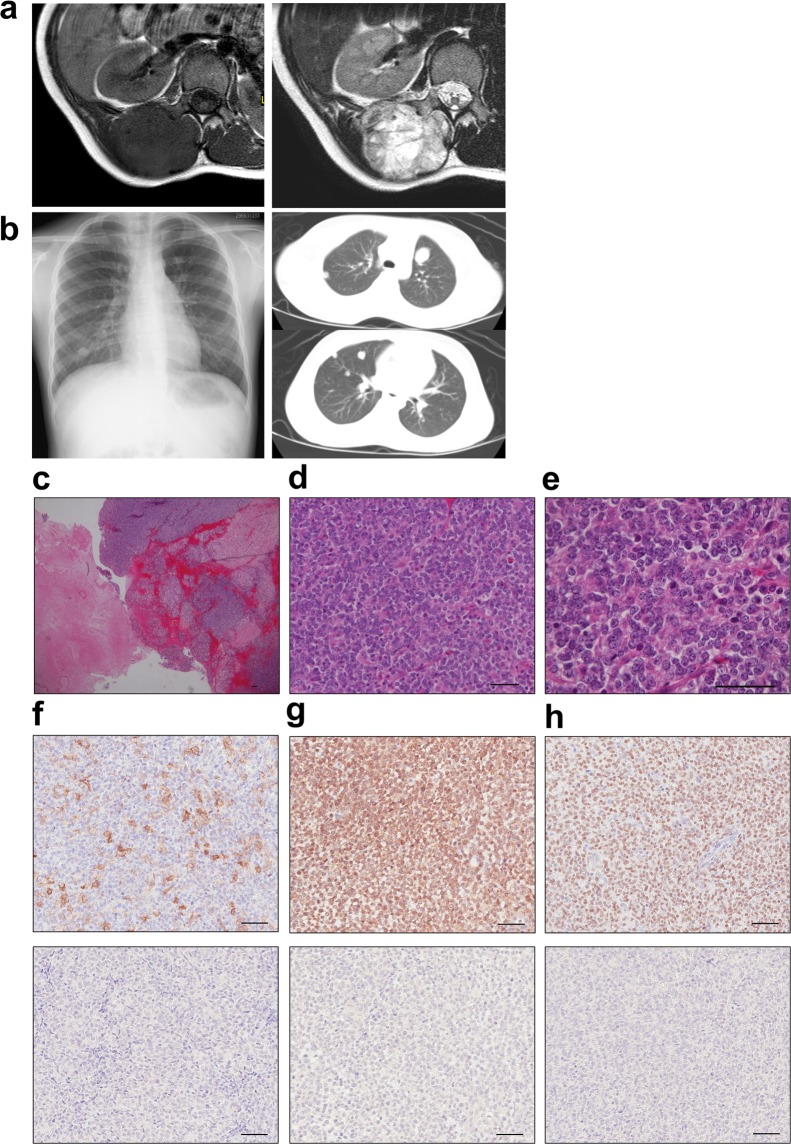
Appearance of the primary tumour in the back and lung metastatic lesions. (**a**) T1- and T2-weighted magnetic resonance images of the primary tumour. (**b**) X-rays and computed tomography of lung metastases. (**c**–**e**) HE staining of the primary tumour tissue. (**f**–**h**) Immunohistochemical analysis of CD99 (**f**), Bcl-2 (**g**), WT1 (**h**) and each negative control. Scale bars: 50 µm.

A 9-year-old girl who presented with more than a 3-month history of a large, painless mass in her back was referred to our institution. Magnetic resonance imaging revealed a huge mass (90 × 50 × 40 mm) that extended from the paravertebral muscle to the epidural space (Fig. 1a). Multiple lung metastases were identified on a plain radiograph and computed tomography at the first presentation to our hospital (Fig. 1b). An open biopsy was performed. Microscopic examination revealed that the tumour was composed of uniform small round cells with round nuclei, with massive necrotic and haemorrhagic lesions (Fig. 1c) and frequent mitotic figures (Fig. 1d,e). Immunohistochemical analyses showed focally positive staining for CD99 (Fig. 1f) and diffusely positive staining for Bcl-2 and WT1 (Fig. 1g,h). On the day after the biopsy, because of the sudden occurrences of leg weakness, loss of sensation, and urinary retention, radiation therapy followed by combination chemotherapy of vincristine, doxorubicin, cyclophosphamide, ifosfamide, and etoposide was immediately begun. After seven courses of this chemotherapy regimen, the primary tumour in the back was surgically resected, and she underwent two courses of adjuvant chemotherapy consisting of vincristine, ifosfamide, and actinomycin D. Three months after the initial surgery, she underwent a lower left lobectomy and lower right partial lobectomy of the lungs. She was treated with two courses of irinotecan and carboplatin, and then melphalan with peripheral blood stem cell transplantation. Nevertheless, the tumour recurred in the right chest wall 4 months after the second operation. The tumour did not respond to either pazopanib or successive lines of irinotecan and temozolomide. Despite the multidisciplinary treatment, the patient died of her disease 16 months after the initial diagnosis.

### Phenotypic characterization and transcriptome profiling of Kitra-SRS cells

**Figure 2:**
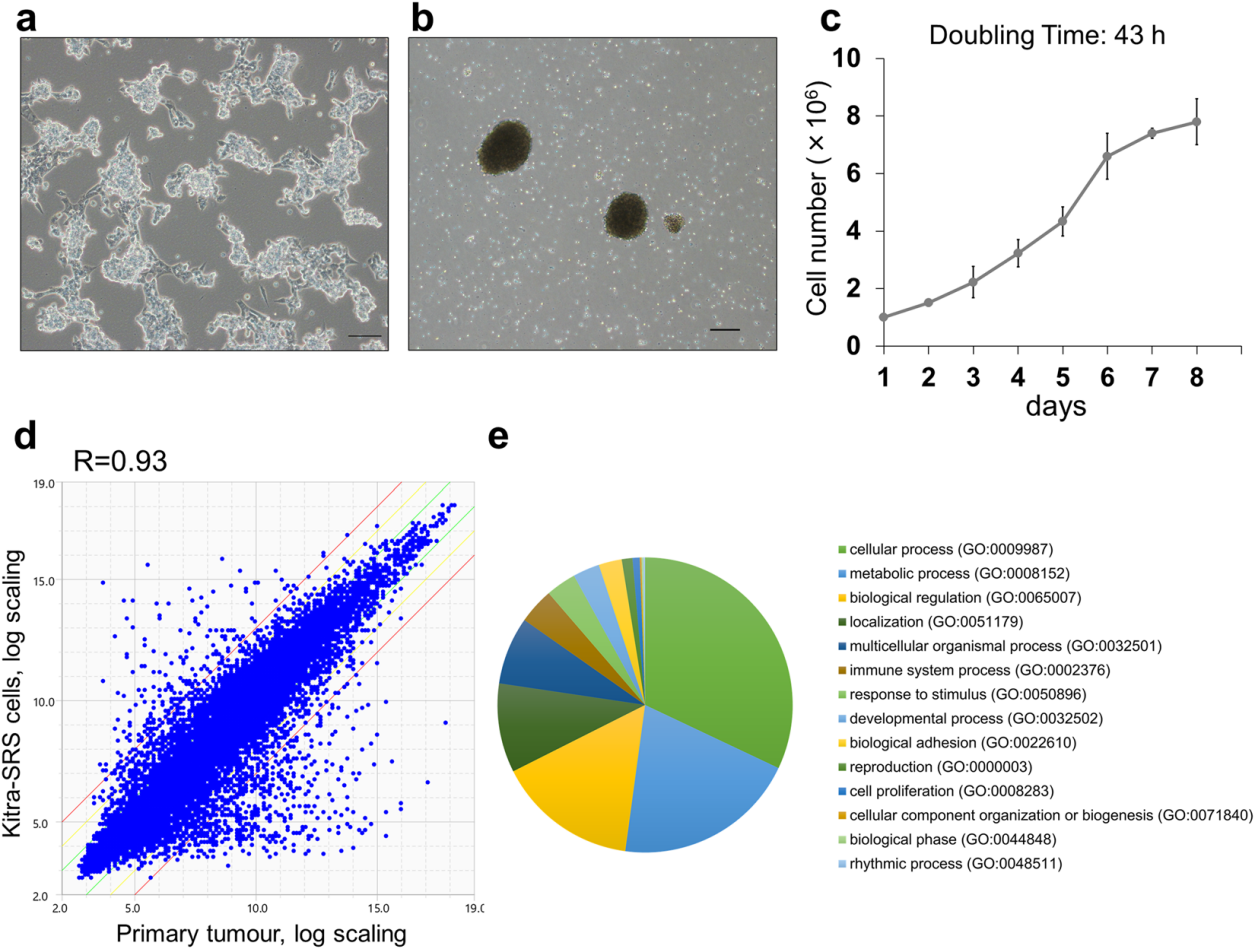
Phenotypic characteristics and gene expression signatures of Kitra-SRS cells. (**a**,**b**) Morphology of Kitra-SRS cells cultured in 2D standard plates (**a**) and in ultra-low attachment plates (**b**) assessed with phase-contrast microscopy. Scale bars: 100 µm. (**c**) Growth curve of Kitra-SRS cells *in vitro*. Each point represents the mean value ± SD (n = 3). (**d**) A scatter plot showing the correlation of gene expression between Kitra-SRS cells and the primary tumour. Expression values are derived from transcriptome analysis of two biological replicates. R represents the correlation coefficient. Green and red lines indicate absolute log-fold-change >1 and >3, respectively. (**e**) A pie chart depiction of PANTHER-GO slim biological processes that had more than 4-fold gene expression change.

The Kitra-SRS cell line has now been growing in standard 2D culture for more than 100 passages (more than 24 months). Kitra-SRS cells were prone to piling up in 2D culture conditions (Fig. 2a). In addition, Kitra-SRS cells showed high anchorage-independent growth ability and formed tumour spheres on low-attachment dishes; they did not form well-rounded structures (Fig. 2b). The doubling time of Kitra-SRS cells in 2D culture conditions in the logarithmic growth phase was approximately 43 h (Fig. 2c). Short tandem repeat (STR) patterns of alleles were identical in the primary tumour, and distinct from any other cell lines in the Japanese Collection of Research Bioresources Cell Bank (JCRB, http://jcrbcelldata.nibiohn.go.jp) and the Cellosaurus database (https://web.expasy.org/cellosaurus-str-search/) (Supplementary Table S1).

Next, gene expression profiles of Kitra-SRS cells and the primary tumour were obtained by transcriptome analysis (Supplementary Dataset 1). Differences in gene expression signatures between them were greater than differences between each sample (Fig. 2d, Supplementary Fig. S1). The transcriptome analysis showed that 1625, 273 and 79 genes were upregulated in Kitra-SRS cells compared to the primary tumour at fold changes >2, >4 and >8, respectively (Supplementary Dataset 2). In addition, 764, 203 and 80 genes were down-regulated in the same criteria, respectively (Supplementary Dataset 3). We also analysed 476 genes with more than 4-fold expression fluctuation for functional and ontological characterization via PANTHER tools (http://www.pantherdb.org). The pie chart of PANTHER-GO slim biological processes showed enrichment of cellular process (164 genes), metabolic process (103 genes), biological regulation (79 genes), localization (50 genes) and multicellular organismal process (38 genes) (Fig. 2e, Supplementary Dataset 4). These results suggested that gene expression signatures of Kitra-SRS cells were not completely identical to those of the primary tumour.

### Identification of the *CIC-DUX4* fusion transcript in Kitra-SRS cells

**Figure 3:**
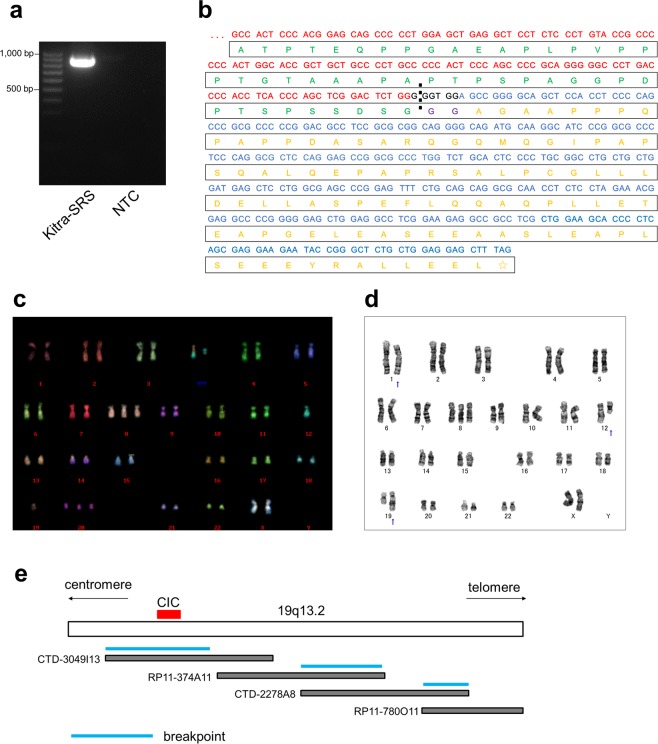
Genetic analysis of Kitra-SRS cells. (**a**) RT-PCR with the *CIC* forward primer located in exon 16 and the *DUX4* reverse primer in exon 1. No band is present for the negative control (NTC) of distilled water in lane 3. (**b**) Nucleotide and predicted amino acid sequences of the *CIC-DUX4* fusions. Two additional amino acid residues that do not come from either *CIC* or *DUX4* are present at the fusion point. Red indicates the *CIC* nucleotide sequence; blue, *DUX4* nucleotide sequence; black, nucleotide sequence not belonging to *CIC* or *DUX4*; green, CIC amino acid sequence; yellow, DUX4 amino acid sequence; purple, amino acid sequence not belonging to CIC or DUX4. (**c**) A representative karyotype of Kitra-SRS cells at passage 20. M-FISH analysis showed four recurrent structural chromosomal rearrangements: 48, XX, del(1)(p32), +8, t(12;19) (q13;q13), +20. (**d**) A representative karyotype of Kitra-SRS cells at passage 100. G-banding showed four recurrent structural chromosomal rearrangements: 47, XX, del(1)(p?), +8, der(12)add(12)(p13)t(12;19) (q13;q13.1), der(19) t(12;19) (q13;q13.1). (**e**) A physical map of 19q13.2 and bacterial artificial chromosome clones used for identification of breakpoints.

To investigate whether Kitra-SRS cells harboured oncogenic fusion genes, high-throughput RNA-seq using fusion discovery algorithms was carried out. Importantly, the *CIC-DUX4* fusion transcript was detected in Kitra-SRS cells (Supplementary Table S2). Reverse transcription polymerase chain reaction (RT-PCR) analysis of Kitra-SRS cells was then performed to check for chimeric *CIC-DUX4* transcripts using a combination of the CIC4120 forward primer and DUX4Tr2 reverse primer (Supplementary Table S3)^21^. As depicted in Fig. 3a, lane 2, *CIC-DUX4* fusions were observed in Kitra-SRS cells. Furthermore, the full-length *CIC-DUX4* cDNA was isolated from Kitra-SRS cells by RT-PCR and subcloned into the pENTR 1A Dual Selection Vector. Sequence analysis revealed that the *CIC* and *DUX4* breakpoint in Kitra-SRS cells was coincident with the insertion of six nucleotides and was confirmed within exon 20 of *CIC* and exon 1 of *DUX4*, respectively, suggesting that Kitra-SRS cells shared the same *CIC* breakpoint as the formerly published findings (Fig. 3b)^21^. Moreover, the *CIC* sequence of the fusion transcript corresponded to the wild-type sequence, and the *DUX4* sequence was identical to sequences of several *DUX4* pseudogene components on chromosomes 4q35.2 or 10q26.3 (Fig. 3b, Supplementary Table S4). Based on the cDNA sequence analysis results, the amino acid sequence of the chimeric protein was predicted (Fig. 3b). The deduced chimeric protein formed an in-frame fusion between CIC and DUX4 with the *CIC* open reading frame and the *DUX4* stop codon. Two additional glycine residues were present at the fusion point, which did not belong to native CIC or *DUX4*.

In chromosomal analysis employing multiplex fluorescence *in situ* hybridization (M-FISH), six out of ten metaphase cells from Kitra-SRS cells at passage 20 showed the following karyotype: 48, XX, del(1)(p32), +8, t(12;19)(q13;q13), +20 (Fig. 3c, Supplementary Table S5). Besides, the karyotype of Kitra-SRS cells at passage 100 was also examined by G-banding. In 15 out of 20 metaphase cells, the karyotype was found: 47, XX, del(1)(p?), +8, der(12)add(12)(p13)t(12;19)(q13;q13.1), der(19)t(12;19)(q13;q13.1) (Fig. 3d, Supplementary Table S6), suggesting a possibility of the alteration of chromosomal abnormalities due to continuous culturing.

Notably, three chromosome breakpoints within 19q13.2 were demonstrated using the bacterial artificial chromosome cloning system, located within the bacterial artificial chromosome clones CTD-3049I13, RP11-374A11 (or CTD-2278A8), and RP11-780O11 (or CTD-2278A8) (Fig. 3e, Supplementary Fig. S2). CTD-3049I13 covered the approximately 200-kb region within 19q13.2 and contained *CIC*. These results suggest that complex chromosomal rearrangements occurred in Kitra-SRS cells with insertion of a chromosome region including a *DUX4* pseudogene after the breakpoint of *CIC* on chromosome 19q13, resulting in generation of the *CIC-DUX4* fusion gene.

### Kitra-SRS cells exhibit tumourigenicity and lung metastasis in nude mice

**Figure 4:**
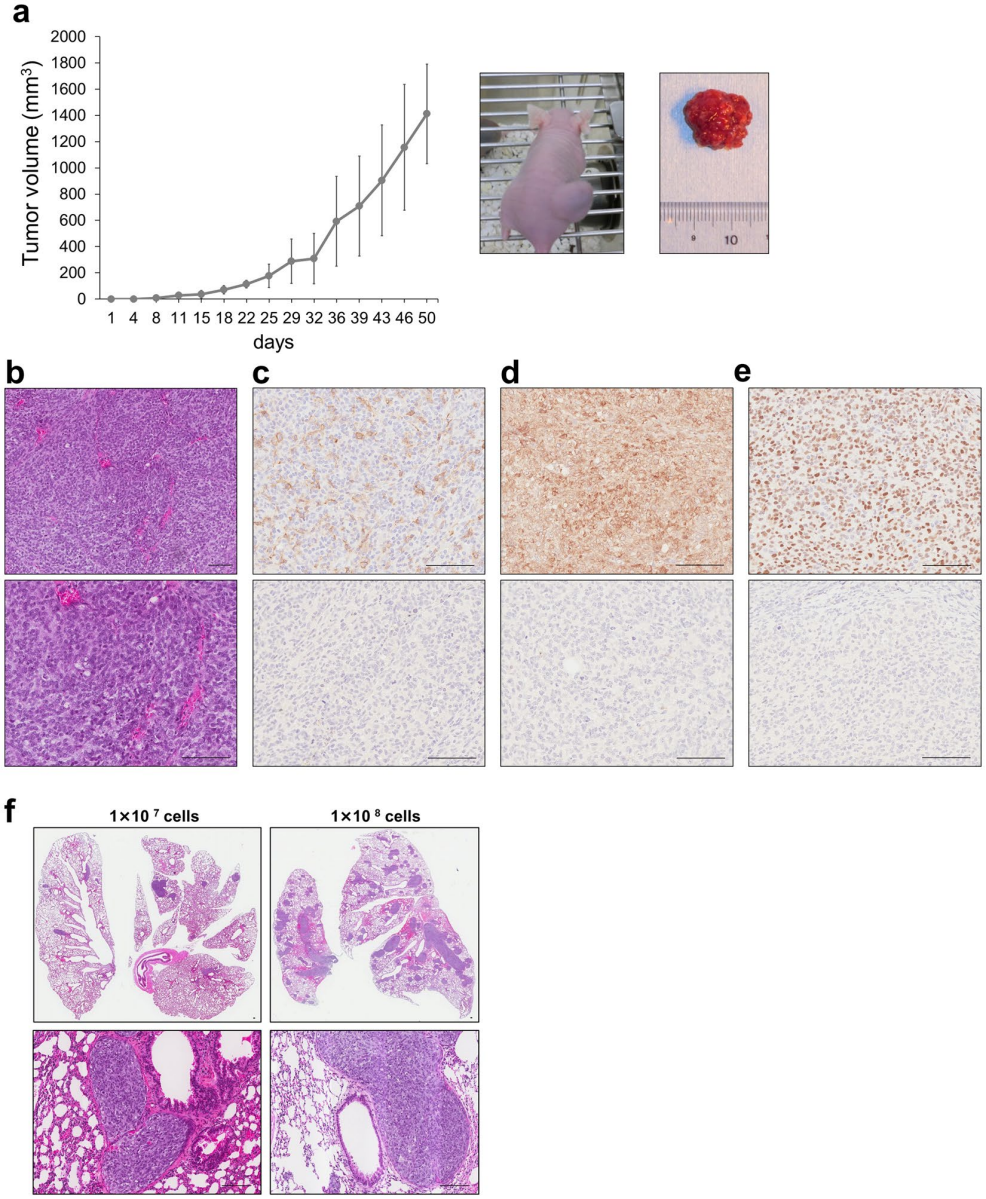
Characteristics of Kitra-SRS tumours *in vivo*. (**a**) Growth curve of Kitra-SRS tumours in nude mice. Each point represents the mean value ± SD (n = 4). (**b**) HE staining of Kitra-SRS tumours. (**c**–**e**) Immunohistochemical analysis of CD99 (**c**), Bcl-2 (**d**) and WT1 (**e**) and each negative control. (**f**) HE staining of lung sections in mice inoculated with 1 × 10^7^ or 1 × 10^8^ Kitra-SRS cells. Scale bars: 100 µm.

To assess tumourigenicity of Kitra-SRS cells *in vivo*, 1 × 10^7^ Kitra-SRS cells were subcutaneously injected into the dorsal flank of five nude mice. Four out of five mice developed solid tumours at the sites of injection. The average tumour volume reached around 1,400 mm^3^ 7 weeks after injection (Fig. 4a). In hematoxylin and eosin (HE)-stained sections of xenografts, tumour cells had round hyperchromatic nuclei, prominent nucleoli, and scanty clear cytoplasm (Fig. 4b). The morphological features of these tumours were similar to those observed in the original tumour. Consistent with the primary tumour, immunohistochemical analyses also revealed focal positivity for CD99 and diffuse positivity for Bcl-2 and WT1 (Fig. 4c–e).

Next, to examine whether Kitra-SRS cells spread to the lungs, 1 × 10^7^ or 1 × 10^8^ Kitra-SRS cells were injected subcutaneously into the back of nude mice (n = 5 each). Intriguingly, 7 weeks after subcutaneous inoculation, pulmonary metastases were clearly observed in four out of five and three out of five Kitra-SRS-inoculated mice, respectively (Supplementary Table S7). The metastatic lung tumours displayed the same histological appearance as the xenografts (Fig. 4f). These findings suggest that Kitra-SRS cells have a high capacity for tumourigenesis and lung metastasis.

### The IGF-1R pathway is constitutively hyperactivated in Kitra-SRS cells

**Figure 5:**
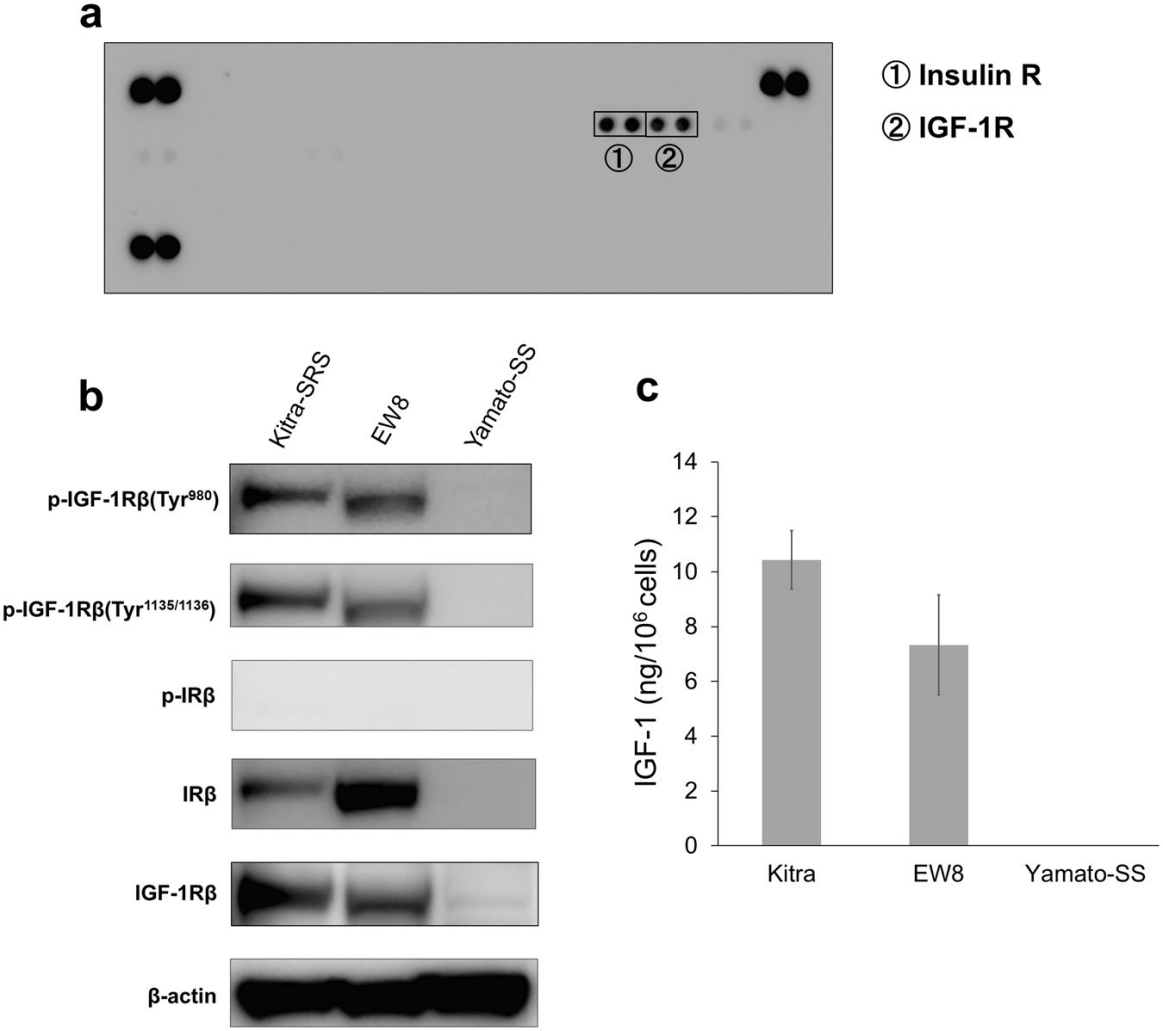
The IGF-1/IGF-1R pathway is activated in Kitra-SRS cells. (**a**) Phospho-RTK array analysis of Kitra-SRS cells. (**b**) Phosphorylation of IGF-1R and IR in Kitra-SRS, EW8, and Yamato-SS cells was assessed with western blotting. (**c**) Amount of IGF-1 secretion by these cells. Data in a bar graph represents the mean value ± SD, n = 3.

To elucidate the potential RTKs that are crucial for cell proliferation in Kitra-SRS cells, we performed phospho-RTK array analysis. Phosphorylation of insulin receptor (IR) and IGF-1R was observed in Kitra-SRS cells (Fig. 5a). Immunoblotting analyses were then conducted to validate activation of these receptors in Kitra-SRS cells. IGF-1R was consistently activated in Kitra-SRS cells as well as in EW8 cells, and IR was inactivated (Fig. 5b, full-length blots are presented in Supplementary Fig. S3). Moreover, the amount of IGF-1 secreted into the culture medium of 1 × 10^6^ Kitra-SRS cells after 72 h in culture was high (Fig. 5c), suggesting that autocrine IGF-1 signalling may play critical roles in providing self-sustaining growth signals to Kitra-SRS cells.

### Linsitinib inhibits Kitra-SRS cell proliferation *in vitro*

**Figure 6:**
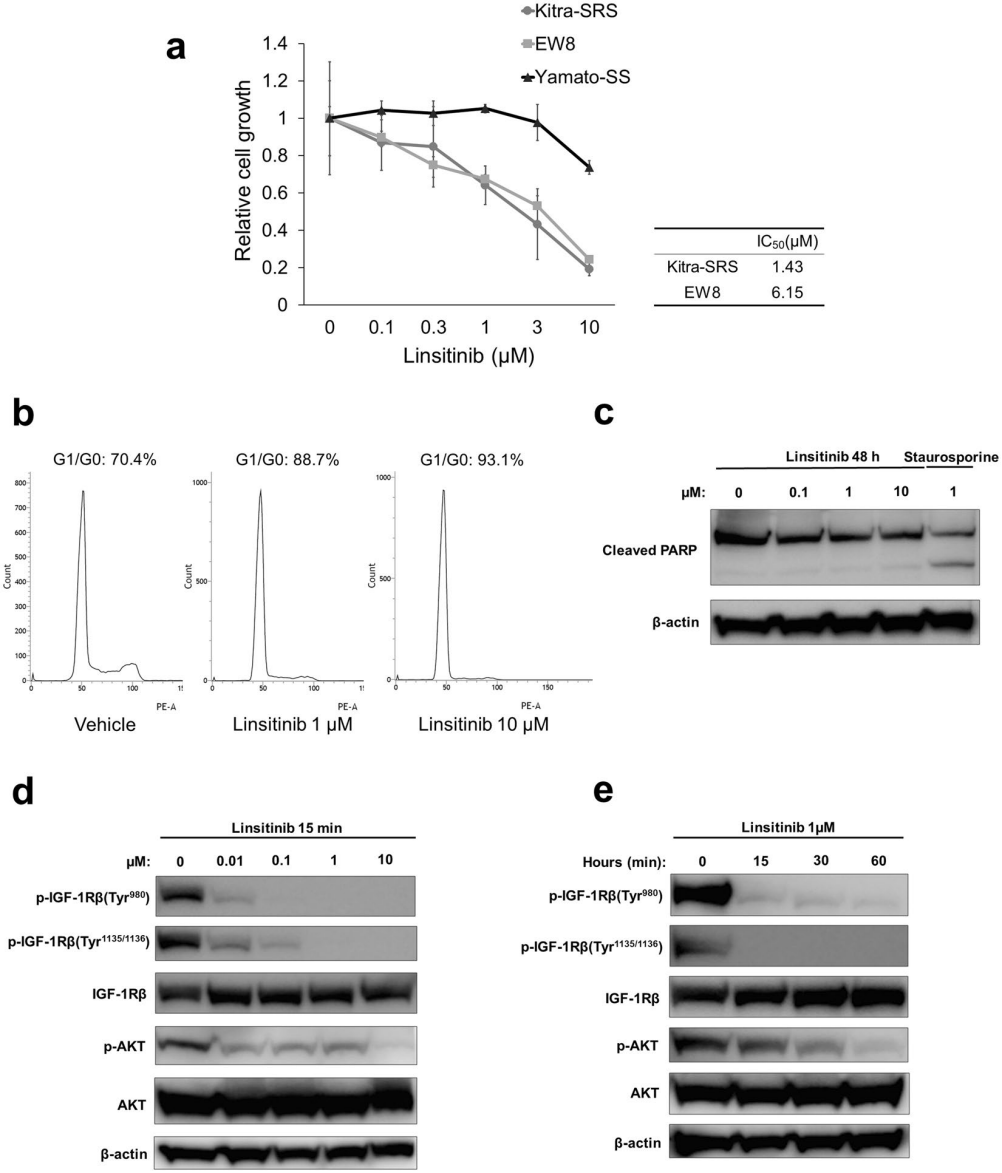
Linsitinib reduced the number of viable Kitra-SRS cells by inducing G1/G0 cell cycle arrest. (**a**) Kitra-SRS, EW8, and Yamato-SS were treated with 0–10 µM linsitinib for 72 h, and the number of viable cells was estimated with the WST-8 assay. The calculated IC_50_ values are shown in the table. Each point represents the mean value ± SD (n = 3). (**b**) Kitra-SRS cells were treated with 0–10 µM linsitinib for 48 h, stained with propidium iodide, and analysed for the cell cycle stage with flow cytometry. (**c**) Kitra-SRS cells were treated with 0–10 µM linsitinib for 48 h, and expression of cleaved PARP was evaluated with western blotting. Staurosporine was used as a positive control. (**d**,**e**) Kitra-SRS cells were treated with 0–10 µM linsitinib for 15 min (**d**) or 1 µM linsitinib for 0–60 min (**e**) and then subjected to western blotting with the indicated antibodies.

The IGF-1R pathway has long been recognized for its role in tumourigenesis and growth of various cancers^22^. Because Kitra-SRS cells exhibited constitutive activation of the IGF-1R pathway, we next assessed the potential anti-proliferative efficacy of the selective IGF-1R inhibitor, linsitinib, which is a potent, orally available small-molecule inhibitor, against Kitra-SRS cells^23^. Linsitinib impaired cell proliferation of Kitra-SRS cells in a dose-dependent manner (Fig. 6a). The 50% inhibitory concentration (IC_50_) value for linsitinib was 1.43 µM. The proliferation of Kitra-SRS cells was highly sensitive to linsitinib, in accordance with the effects on EW8 cells in which IGF-1R signalling is hyperactivated^24^. In addition, flow cytometric analyses revealed that exposure to linsitinib for 48 h increased the population of Kitra-SRS cells in the G0/G1 phase in a dose-dependent manner (Fig. 6b). The cellular level of cleaved poly (ADP-ribose) polymerase (PARP), which are a hallmark of apoptosis, was not activated in Kitra-SRS cells treated with 0–10 µM linsitinib for 48 h, as seen with western blot analyses (Fig. 6c, full-length blots are presented in Supplementary Fig. S4). These experiments suggest that linsitinib causes G0/G1 cell cycle arrest, but not apoptosis in Kitra-SRS cells. We then examined whether linsitinib inhibited activation of IGF-1R and its downstream pathways in Kitra-SRS cells. Linsitinib exposure for 15 min inhibited autophosphorylation of IGF-1R in a dose-dependent manner (Fig. 6d, full-length blots are presented in Supplementary Fig. S5). Additionally, 1 µM linsitinib inhibited phosphorylation of IGF-1R/AKT signalling in a time-dependent manner (Fig. 6e, full-length blots are presented in Supplementary Fig. S6).

### Linsitinib significantly abrogates Kitra-SRS tumour growth in xenograft mouse models

**Figure 7:**
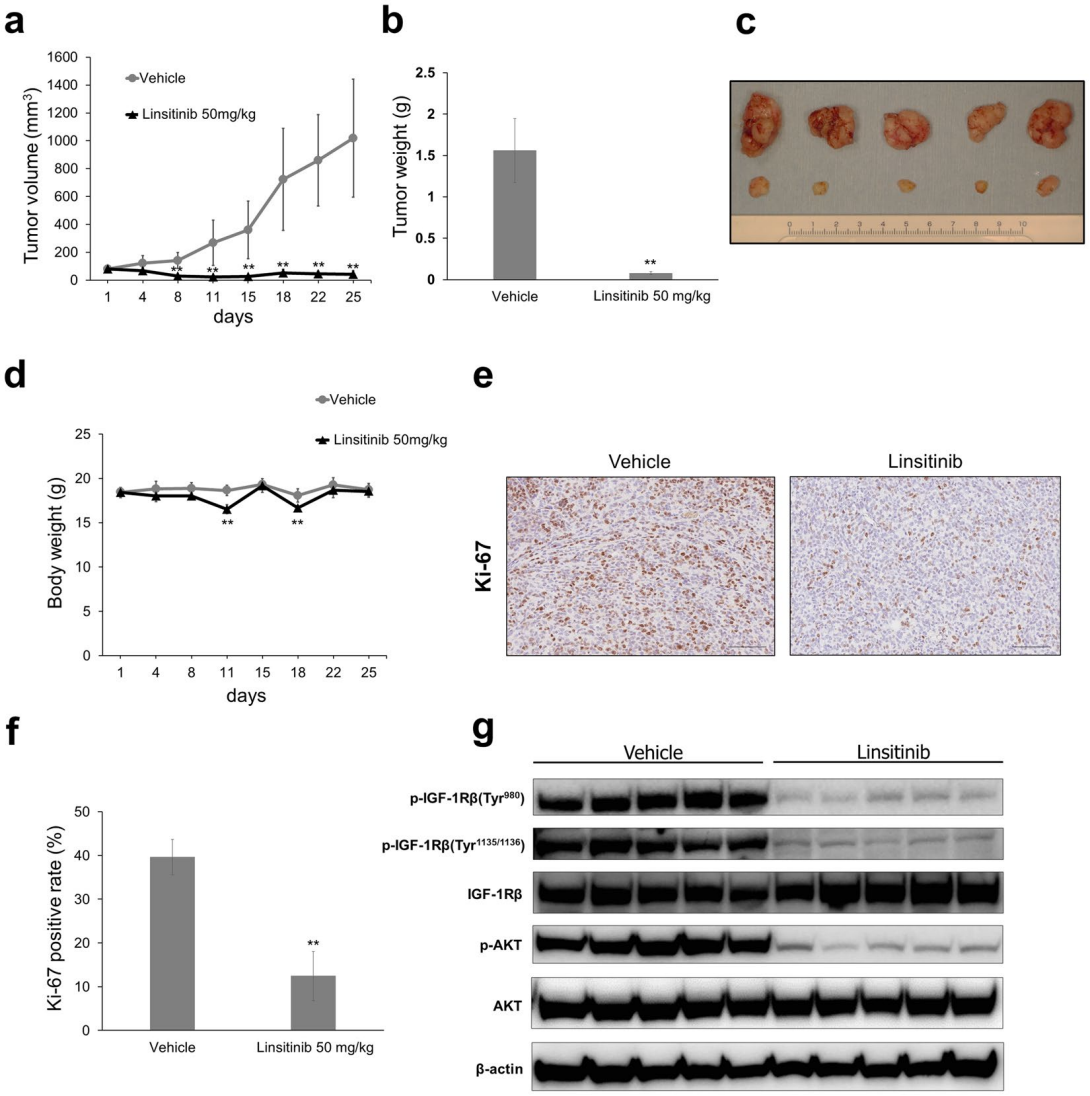
Linsitinib significantly abrogates Kitra-SRS tumour growth in xenograft mouse models. (**a**–**d**) Mice bearing Kitra-SRS xenografts were treated with either 50 mg/kg linsitinib or vehicle (five mice/group). Tumour volume and body weight during treatment (**a**,**d**) and tumour weight and the appearance of resected tumours at the end point (**b**,**c**) are shown. (**e**) Immunohistochemical staining showing expression of Ki-67 in each treatment group. (**f**) Quantification of Ki-67-positive cells in Kitra-SRS tumours from each treatment group (five fields counted/group). (**g**) Tumour tissues from Kitra-SRS xenografts at the end point were harvested, and lysates were prepared for western blot analyses using the indicated antibodies. Data in a bar graph represent the mean value ± SD. **p < 0.01; significantly different from the vehicle group.

Finally, we evaluated the antitumour effects of linsitinib against Kitra-SRS xenograft tumours in nude mice. Mice were injected subcutaneously with Kitra-SRS cells, and tumours were allowed to grow until the average diameter reached 5 mm. Mice were then treated orally with 50 mg/kg linsitinib or with an equal volume of vehicle for 5 days a week for 4 weeks. Treatment with 50 mg/kg linsitinib markedly suppressed tumour growth compared with the vehicle control (Fig. 7a–c). In linsitinib-treated mice, some effects on body weight were observed, with an average loss of 4.1% of body weight during the study (Fig. 7d). At the end of linsitinib treatment (40 days after subcutaneous inoculation), pulmonary metastases were found in all the vehicle controls; on the other hand, none were observed in linsitinib-treated mice (Supplementary Fig. S7). Immunohistochemical analyses revealed that fewer cells in linsitinib-treated mice were immunopositive for the proliferating cell marker, Ki-67, than in vehicle-treated mice (Fig. 7e,f, a negative control is presented in Supplementary Fig. S8). Tumour lysates extracted from Kitra-SRS xenografts were also investigated for IGF-1R/AKT signalling. Consistent with *in vitro* data, linsitinib treatment markedly decreased phosphorylation of IGF-1R/AKT signalling (Fig. 7g, full-length blots are presented in Supplementary Fig. S9). These results demonstrated that linsitinib suppressed Kitra-SRS tumour growth by suppressing cell cycle progression, at least in part, through inhibition of IGF-1R/AKT signalling.

### Drug screening in Kitra-SRS cells

**Figure 8:**
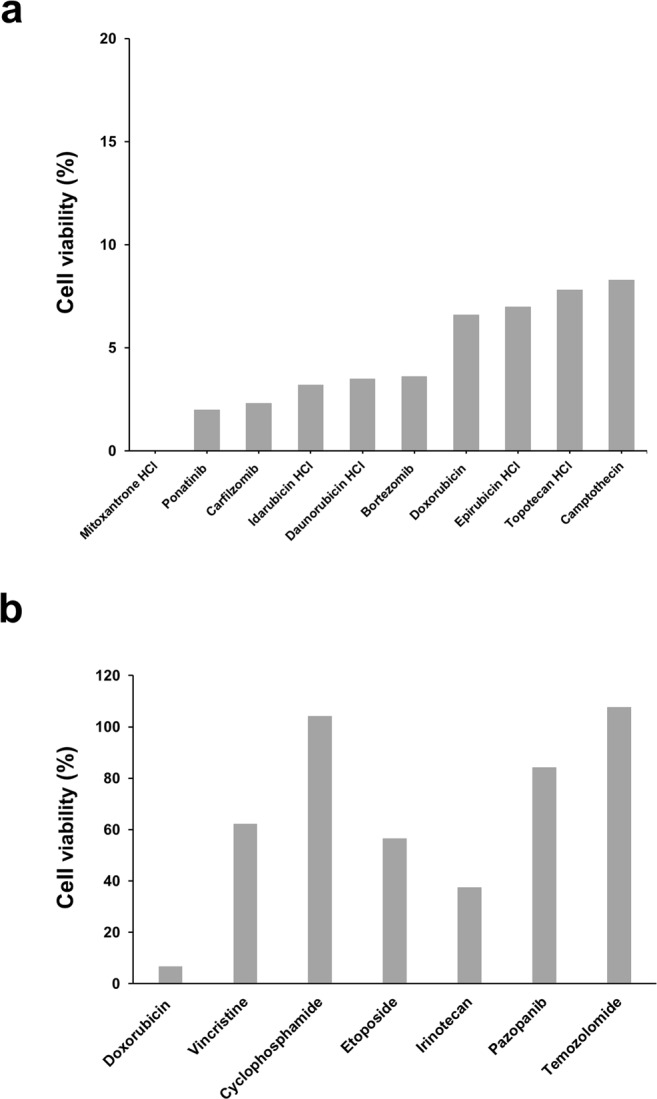
Screening of 1134 FDA-approved drugs in Kitra-SRS cells. Ten micromolar of 1134 FDA-approved drugs in our library were added to Kitra-SRS cells for 48 h. (**a**) Ten anticancer drugs that inhibited more than 80% cell viability in Kitra-SRS cells. (**b**) The antiproliferative efficacy of the drugs used in the donor patient.

To examine sensitivity to treatments with various drugs in Kitra-SRS cells, we screened 1134 FDA-approved drugs at a fixed concentration (10 µM) (Supplementary Dataset 5). Selective IGF-1R inhibitors were not included in them. Seventeen drugs exhibited remarkable antiproliferative effects, which were defined as a loss of >80% cell viability in Kitra-SRS cells (Supplementary Table S8). Among them, ten drugs were used as anticancer drugs (Fig. 8a). Doxorubicin, which was used for the donor patient treatment, was one of the most effective drugs in Kitra-SRS cells. However, the other chemotherapy reagents used in the donor patient such as vincristine, cyclophosphamide, etoposide, irinotecan, pazopanib and temozolomide did not exhibit potent cytotoxic effects on Kitra-SRS cells (Fig. 8b). Indeed, the lung metastases of the patient showed a partial response to VDC-IE (vincristine, doxorubicin, cyclophosphamide, ifosfamide and etoposide) chemotherapy. Consistent with *in vitro* data, pazopanib, irinotecan and temozolomide were largely ineffective in the donor patient at advanced stage. These results suggest that the responses of Kitra-SRS cells to chemotherapy reagents are likely to reflect those of the primary tumour.

## Discussion

Cancer cell lines and xenograft models are essential tools for uncovering the biologic processes in oncology and discovering novel therapeutic strategies. Regarding CDS, to our best knowledge, only three cell lines (ECD1, NCC-CSD-X1-C1, and NCC-CDS1-X3-C1) and two patient-derived xenografts (NCC-CSD-X1 and NCC-CDS1-X3) are available that can be used for preclinical research^7,20^. In the present study, we succeeded in establishing the first human CDS cell line, Kitra-SRS, that can be persistently cultured *in vitro* and xenografted into nude mice. Kitra-SRS cells exhibited metastatic potential to lungs in the xenograft mouse model. Additionally, we demonstrated that the growth of Kitra-SRS cells predominantly depended on activated IGF-1R/AKT signalling in an autocrine manner.

We conducted comparative transcriptome analysis of Kitra-SRS cells and the primary tumour, which indicated that their expression patterns were not completely identical. Our data is consistent with the result of a global gene expression study showing that cell lines may not perfectly conserve the molecular features of original tumour cells *in vivo*^25^. In addition, the pathway-specific differences between *in vitro* and *in vivo* tumor cells were previously reported at the gene expression level^26^. One reason for these differences is likely that the conditions of tissue culture cells are quite different from those of tumour cells *in vivo*. Another reason is likely that normal cells in the tumour tissues such as stromal cells, immune cells and endothelial cells are removed during the process of cell line establishment. These observations suggest that although cell lines and mouse xenograft models are useful research resources, they may not stand for a global model of clinical tumours. From this point of view, it would be helpful to compare multiomics data, such as genome, transcriptome and proteome data, of cell lines with those of the primary tumours in order to make the best use of cell lines.

The *CIC-DUX4* fusion gene has been suggested to result from either a t(4;19)(q35;q13) or t(10;19)(q26;q13) translocation^1,7,14,21,27^. However, Antonescu *et al*. recently used FISH and documented the clinicopathologic features of a large cohort of 115 cases with a *CIC* break-apart signal; only 65 cases were positive for fusion to *DUX4* on 4q35 or 10q26; in the remaining 50 cases, a fusion partner could not be identified^28^. Our results may partially explain these findings; we showed that a *DUX4* pseudogene component was inserted in exon 20 of *CIC* along with t(12;19) complex chromosomal rearrangements in Kitra-SRS cells. Therefore, the *CIC-DUX4* fusion is likely generated by a variety of chromosome rearrangements including translocations and insertions.

The presence of multiple *CIC-DUX4* fusion variants has been reported^29,30^. Consistent with our results, the breakpoints of *CIC* are mostly located within exon 20, leaving intact the functional regions of *CIC*, including the N-terminal DNA-binding high-mobility group box and C-terminal C1 domains^29,31,32^. Meanwhile, *DUX4* breakpoints are scattered throughout exons 1–2 or intron 1, although no significant differences were found in a comparison of clinicopathological data and fusion points between *CIC* and *DUX4*^29,30^. Kitra-SRS cells exhibited wild-type *CIC* fused to a *DUX4* pseudogene that was identical to several *DUX4* pseudogene sequences. Because *DUX4* pseudogenes have high sequence homology on both chromosomes 4q35.2 and 10q26.3, a distinction between *DUX4* pseudogenes could not be made. In prior reports, some cases showed *CIC* fused to the 3′-untranslated region of *DUX4*, resulting in a stop codon after only a few amino acids, suggesting that these cases were not predicted to generate a chimeric protein with a significant *DUX4* component. Moreover, *CIC* fusion to non-*DUX4* genes such as *FOXO4*, *NUTM1*, and *NUTM2A* seems to lead to histology and features similar to those with *CIC-DUX4*, although *CIC-LEUTX* sarcomas are related to CD31 and ETS-related gene expression^1,3–6,8^. Loss-of-function of CIC is a key molecular event in multiple human cancers^33,34^. Thus, C-terminal truncated CIC may play a key role in the oncogenesis of CDS.

The diagnosis of CDS requires identification of *CIC* rearrangement. *CIC-DUX4* fusion transcripts can be demonstrated in clinical practice with FISH and the *CIC* break-apart strategy, which should, in theory, detect every *CIC*-rearranged sarcoma. However, the FISH false-negative rate for *CIC*-rearranged sarcomas is 14–46%^9,29,30^. RNA-seq is also a useful tool for validating *CIC-DUX4* fusion genes. In the present study, we discovered *CIC-DUX4* in Kitra-SRS cells with RNA-seq using fusion discovery algorithms. However, Kao *et al*. reported 14 cases lacking genetic abnormalities as assessed by RNA-seq but exhibiting *CIC-DUX4* fusion genes as determined by FISH and manual inspection of *CIC* sequences^30^. These results suggest the consistent failure of *CIC-DUX4* identification by RNA-seq as well as FISH. Immunohistochemical analysis of ETV4 and WT1 are also helpful ancillary techniques for diagnosing CDS^2,9–11^. Kitra-SRS cells showed positivity for CD99, Bcl-2 and WT1. CD99 and WT1 is positive in most CDS patients; however, WT1 is more specific marker than CD99, which was reported to be 95% sensitive and 81% specific among CDS^11,14^. Since *Bcl-2* was upregulated in an *ex vivo* mouse model for CDS by transducing embryonic mesenchymal cells with *CIC-DUX4*, it is likely to be a *CIC-DUX4* downstream gene albeit non-specific marker for CDS^15^. Taken together, for a more appropriate diagnosis of CDS, judicious use of multiple modalities such as FISH, RNA-seq, or histology including immunohistochemistry is necessary.

Our data indicated that the aggressive nature of Kitra-SRS cells was attributed, in part, to hyperactivation of IGF-1R/AKT signalling. Since IGF-1R signalling is also activated in small round cell sarcomas including Ewing sarcomas and rhabdomyosarcomas, these findings raise the possible relationship between the tumor morphology and activated signalling pathway in sarcomas^35–38^. IGF-1R pathways have been implicated in tumourigenesis, metastasis, and resistance to existing forms of cancer therapy in various cancers^39,40^. Several clinical trials have been conducted to evaluate the efficacy of IGF-1R inhibition in Ewing sarcoma patients^38,41–46^. In addition, prior reports demonstrated that the activated IGF-1 pathway is modulated by EWSR1/FLI-1 through transcriptional repression of *insulin-like growth factor binding protein 3*, a negative regulator of IGF-1/IGF-1R signalling^47–49^. Thus, the correlation between the *CIC-DUX4* fusion protein and IGF-1/IGF-1R signalling in Kitra-SRS cells is intriguing; in this study, however, the relationship remains unclear. Activation of IGF-1R signalling may be derived from the potential cell of origin for Kitra-SRS cells. Further investigation is required for definitive answers to this challenging issue. This exploration and resolution may not only shed light on the process of oncogenesis but may also lead to potentially novel targets for CDS therapy.

By screening a library of 1134 FDA-approved drugs, we found that ten anticancer drugs exerted remarkable antiproliferative effects against Kitra-SRS cells. Although seven out of them are DNA intercalators, which inhibit DNA replication by interacting with DNA, three drugs such as ponatinib, carfilzomib and bortezomib have different pharmacological activities. Ponatinib is an inhibitor for multi-tyrosine kinases such as SRC, ABL, FGFR, PDGFR and VEGFR^50^. Carfilzomib and bortezomib are proteasome inhibitors^51,52^. Our findings corresponded to those previously reported by Oyama *et al*., which showed growth-suppressive effects of DNA intercalators, a multi-tyrosine kinase inhibitor (crizotinib) and a proteasome inhibitor (bortezomib) on two CDS cell lines (NCC-CSD-X1-C1 and NCC-CDS1-X3-C1)^20^. Since these anticancer drugs are used in clinical practice, they appear to be worth further investigating for clinical trials.

Prospective clinical trials have not yet demonstrated the potential efficacy of individualized chemotherapy selected *in vitro* drug sensitivity testing compared with empiric regimens for patients with cancer^53^. The discordance in the response to drug treatments between *in vitro* and *in vivo* tumor cells may be due to differences in gene expression and surrounding environment. However, in the current study, the sensitivity to anticancer drugs administered to the donor patient seemed to be similar in Kitra-SRS cells and the original tumour. Cell lines with clinical and pathological data are useful resources for interpretation of experimental results because the cell lines deposited in cell banks generally are not supplemented with clinical information about the donor patients. Barretina *et al*. reported that genomic profiles obtained by the Cancer Cell Line Encyclopedia predicted drug sensitivity, suggesting that cell lines would be useful in designing cancer clinical trials^54^. Rees *et al*. also reported that the basal gene expression patterns were linked to those of small- molecule sensitivity across a lot of cell lines^55^. Thus, our model that reproduce the response to treatments with some fidelity would be valuable for development of anticancer drugs and efficient clinical trials.

Intriguingly, it is possible that the orthotopic xenograft tumours engrafted with Kitra-SRS cells metastasized to the lungs spontaneously. In general, it is rare for xenograft tumours to develop lung metastases in nude mice, although some epithelial tumour cell lines and a few sarcoma cell lines such as osteosarcoma and malignant fibrous histiocytoma have been reported to metastasize to lungs in nude mice^56–58^. The primary tumour of Kitra-SRS cells had high lung metastatic potential in the donor patient. Moreover, lung metastases observed in Kitra-SRS xenografts were scattered in the lungs, which appear to be very analogous to those seen in clinical practice. However, in this study, the mechanism of metastases in Kitra-SRS xenografts remains to be elucidate; therefore, further studies are warranted.

In conclusion, the present study describes the establishment and characterization of a human CDS cell line, Kitra-SRS cells, with autocrine activation of IGF-1/IGF-1R signalling and metastatic potential to the lungs. Kitra-SRS will be a meaningful model for investigating the molecular pathology of CDS and developing novel strategies to treat patients with CDS.

## Methods

### Establishment of the Kitra-SRS cell line

Tumour cells were isolated from a portion of the initial surgically resected tissue with informed consent from the patient’s parents and according to the guidelines of the Institutional Review Board for Clinical Research at Osaka University Hospital. The tumour tissues were minced and incubated with 1 mg/mL collagenase type I (Wako, Osaka, Japan) for 1 h at 37 °C. The cells were passed through a 40-µm nylon mesh (BD Falcon, Franklin Lakes, NJ, USA) and cultured in Dulbecco’s modified Eagle’s medium (DMEM; Nacalai Tesque, Tokyo, Japan) supplemented with 100 µg/mL streptomycin sulfate, 100 U/mL penicillin G (Life Technologies, Carlsbad, CA, USA), and 10% heat-inactivated fetal bovine serum (FBS) (Life Technologies) at 37 °C in a humidified atmosphere of 5% CO_2_. The adherent cells have been maintained for over 24 months in culture and have been passaged more than 100 times. Throughout the establishment of this cell line, the attached cells continuously expressed the *CIC-DUX4* transcript. This cell line was designated Kitra-SRS. All methods were performed in accordance with the relevant guidelines and regulations and were approved by the Ethics Committee of Osaka University Graduate School of Medicine (No. 11044-4).

### Cell culture

We utilized EW8 and Yamato-SS cell lines. The EW8 cell line was purchased from the American Type Culture Collection. The Yamato-SS cell line was established from a primary tumour of a patient with synovial sarcoma in our laboratory^59^. Cells were maintained under the same conditions as Kitra-SRS cells.

### Compounds

Linsitinib was purchased from LC Laboratories (Woburn, MA, USA), prepared in dimethyl sulfoxide before addition to cell cultures for *in vitro* examinations and diluted in 25 mM tartaric acid to the desired concentration for *in vivo* experiments.

### Cell proliferation assay

Cells were seeded in six-well plates, and the number of viable cells was counted with a Countess^TM^ automated cell counter (Life Technologies). The doubling time was calculated according to the formula: Doubling time (h) = ln(2)/[ln(cell count_t2_/cell count_t1_)/(T2-T1)], where T2 and T1 represent two distinct time points (h) in the logarithmic culture growth phase. Kitra-SRS, EW8, Yamato-SS cells were seeded in 96-well plates at 2 × 10^4^, 5 × 10^3^, or 5 × 10^3^ cells/well, respectively, in triplicate. Proliferation was measured with Cell Counting Reagent SF (Nacalai Tesque), which is a WST-8 assay system, according to the manufacturer’s instructions. Absorbance was measured using a spectrophotometer.

### Authentication and quality control of the established cell line

Genomic DNA was extracted from the tumour tissues or cultured cells using the DNeasy Blood & Tissue Kit (Qiagen, Hilden, Germany). Authentication of Kitra-SRS cells was achieved by examining STRs at 16 loci using STR multiplex assays (PowerPlex® 16 HS System; Promega, Madison, WI, USA). The STR profiles were compared with the Japanese Collection of Research Bioresources Cell Bank and the Cellosaurus database for reference matching.

### RNA-seq experiment

To evaluate gene expression profiles of Kitra-SRS cells and the primary tumour, total mRNA was processed following the whole-transcript sense target labeling assay from Affymetrix and hybridized in Clariom S Array, Human (Affymetrix, Santa Clara, CA, USA). Primary data analysis was performed with Applied Biosystems^TM^ GeneChip® Expression Console^TM^ Softwere ver.1.3.0. We filtered out control probesets and those probesets that seemed to be either not expressed or at such a low level that noise dominates the signal when analyzing gene expression fluctuations. The criteria of the specific genes were absolute log-fold-change >1. To detect fusion genes in Kitra-SRS cells, total mRNA was converted into a library of template molecules. The cDNA sequencing library was prepared from poly-A-containing mRNA using the Illumina® TruSeq™ RNA Sample Preparation Kit (Illumina, San Diego, CA, USA), according to the manufacturer’s instructions. The cDNA library was sequenced using HiSeq. 2000 (Illumina).

### Genetic analysis

Total RNA was purified using the RNeasy Mini Kit (QIAGEN) and reverse transcribed using the ReverTra Ace qPCR RT Master Mix with gDNA Remover (TOYOBO, Osaka, Japan), according to the manufacturer’s instructions. The *CIC-DUX4* fusion transcript was amplified with RT-PCR using KOD-Plus-Neo (TOYOBO). For Sanger sequence analysis, the full-length *CIC-DUX4* cDNA was amplified with the CIC-fl forward primer and DUX4-fl reverse primer, electrophoresed on 1.5% agarose gels, purified using the QIAquick Gel Extraction Kit (Qiagen), subcloned into the Gateway pENTR 1A Dual Selection Vector (Thermo Scientific, Waltham, MA, USA), and sequenced using the BigDye Terminator v3. 1 Cycle Sequencing Kit (Applied Biosystems) on an Applied Biosystems Model 373A DNA sequencing system. The sequences of primers used for Sanger sequence analysis are shown in Supplementary Table S3. BioEdit (http://en.bio-soft.net/format/BioEdit.html) was used for computer analysis of sequence data.

### Chromosomal analysis

M-FISH analysis was performed at passage 20 as previously described^60^. G-banding was carried out at passage 100. Briefly, Kitra-SRS cells were exposed for 16 h to colcemide solution at final concentration of 0.04 µg/ml. Afterwards, the cells were treated with 0.075 M KCl at 37 °C for 25 min and fixed with Carnoy’s solution (3:1 methanol: glacial acetic acid) at room temperature. Cell suspension was dropped onto glass slides, air-dried and treated by pre-warmed 0.005% trypsin solution at 37 °C for 8 minutes. Staining was done using 6% Giemsa solution in Sorensen buffer for 3.5 min. Slides were rinsed in water and air-dried at room temperature. Cytogenic analysis was done using Ikaros software (MetaSystems, Heidelberg, Germany). Chromosomes were classified according to the International System for Human Cytogenetic Nomenclature (ISCN).

### *In vivo* mouse xenograft models

Five-week-old male BALB/c nu/nu mice (SLC, Shizuoka, Japan) were housed at the Institute of Experimental Animal Sciences, Osaka University Medical School, in accordance with guidelines approved by the Institutional Animal Care and Use Committee of the Osaka University Graduate School of Medicine. To determine tumourigenicity or lung metastatic potential, 1 × 10^7^ or 1 × 10^8^ Kitra-SRS cells were subcutaneously inoculated into the back. Tumour volumes were measured twice weekly from the skin using a caliper, and the volume was calculated as (A × B2)/2, where A is the longest diameter and B is the shortest diameter. After 50 days, all mice were euthanized, and lung metastasis was assessed. For the tumour growth assay, when the average diameter of tumours engrafted with 1 × 10^7^ Kitra-SRS cells reached 5 mm, 50 mg/kg linsitinib was orally administered 5 days a week for 4 weeks. After 28 days of treatment, the mice were euthanized. All protocols were approved by the Animal Care and Use Committee of the Osaka University Graduate School of Medicine.

### Histology and immunohistochemistry

Excised xenograft tumours and lungs were fixed in 10% neutral-buffered formalin for 48 h, embedded in paraffin, cut into 3-µm-thick sections, and examined with HE staining and immunohistochemistry as previously described^60^. Antigens were retrieved at 95 °C for 30 minutes in a 10-mM citrate buffer. The primary antibodies are available in Supplementary Table S9. As negative controls, staining was performed with corresponding isotype controls. The number of Ki67 positive cells was counted for five randomly selected fields under the microscope at 20× magnification using Aperio ImageScope viewing software (Leica Biosystems, Wetzlar, Hesse, Germany) according to the manufacturer’s protocol.

### Phospho-RTK array

The phospho-RTK array comprising spotted antibodies for 49 kinase phosphorylation sites was performed using the Proteome Profiler Array Kit (R&D Systems; Minneapolis, MN, USA), according to the manufacturer’s instructions.

### Western blot analysis

Cells were lysed in radioimmunoprecipitation assay buffer (Thermo Scientific) supplemented with 1% protease/phosphatase inhibitor cocktail (Cell Signaling Technology, Inc., Danvers, MA, USA). Protein concentrations were measured using the bicinchoninic acid method (Thermo Scientific). Cell proteins were separated on 4–12% Bis-Tris gels (Life Technologies), transferred to polyvinylidene difluoride membranes (Nippon Genetics, Tokyo, Japan), and immunoblotted as previously described^60^. The primary antibodies are available in Supplementary Table S9.

### Enzyme-linked immunosorbent assay (ELISA)

A total of 1 × 10^6^ cells/well were seeded in six-well plates in triplicate and cultured for 72 h. A Quantikine ELISA Kit Human IGF-1 (R&D Systems) was used in accordance with the manufacturer’s instructions to measure secreted IGF-1 levels in supernatants.

### Flow cytometry

Kitra-SRS (1 × 10^6^ cells per 100 mm dish) were cultured and grown overnight, followed by linsitinib treatment or vehicle. After treatment for 72 h, the cells were harvested and stained with propidium iodide solution (25 µg/mL propidium iodide, 0.03% NP-40, 0.02 mg/mL RNase A, 0.1% sodium citrate) for 30 min at room temperature. For cell cycle analysis, we used a BD FACSVerse™ flow cytometer (BD Bioscience, Franklin Lakes, NJ, USA) according to the manufacturer’s protocol.

### Drug screen

Cells (20000 cells/well) were plated in 384-well culture plates in DMEM containing 10% FBS and cultured overnight at 37 °C in a humidified atmosphere of 5% CO_2_. Ten micromolar of 1134 FDA-approved drugs in our library (Selleck Chemicals, Houston, TX, USA) were added to the cells, using FLUENT high-throughput assay system (TECAN, Männedorf, Switzerland), and cell viability was measured 48 h later with a Cell Counting Kit-8 (Dojindo, Kumamoto, Japan).

### Statistical analysis

All data are expressed as the mean ± standard deviation (SD). Means from biological assays were compared using the Student’s t-test and means from animal experiments using the Mann–Whitney’s U test. Values of P < 0.05 (two-tailed) were considered significant for all tests.

## Supplementary information

Supplementary information

Dataset 1

Dataset 2

Dataset 3

Dataset 4

Dataset 5

## Data Availability

The datasets analysed during the current study are available from the corresponding author on reasonable request.
