# Author Correction: Establishment of a novel human CIC-DUX4 sarcoma cell line, Kitra-SRS, with autocrine IGF-1R activation and metastatic potential to the lungs

**DOI:** 10.1038/s41598-019-55752-0

**Published:** 2020-01-15

**Authors:** Sho Nakai, Shutaro Yamada, Hidetatsu Outani, Takaaki Nakai, Naohiro Yasuda, Hirokazu Mae, Yoshinori Imura, Toru Wakamatsu, Hironari Tamiya, Takaaki Tanaka, Kenichiro Hamada, Akiyoshi Tani, Akira Myoui, Nobuhito Araki, Takafumi Ueda, Hideki Yoshikawa, Satoshi Takenaka, Norifumi Naka

**Affiliations:** 1 Department of Orthopaedic Surgery, Osaka University Graduate School of Medicine, 2-2 Yamadaoka, Suita, Osaka, 565-0871 Japan; 2 Department of Orthopaedic Surgery, Yao Municipal Hospital, 1-3-1 Ryugecho, Yao, Osaka, 581-0069 Japan; 3 Department of Orthopaedic Surgery, Kawachi General Hospital, 1-31 Yokomakura, Higashiosaka, Osaka, 578-0954 Japan; 4 Musculoskeletal Oncology Service, Osaka International Cancer Institute, 3-1-69 Otemae, Chuo-ku, Osaka, 541-8567 Japan; 5 Compound Library Screening Center, Osaka University Graduate School of Pharmaceutical Sciences, 1-6 Yamadaoka, Suita, Osaka, 565-0871 Japan; 6 Department of Orthopaedic Surgery, Ashiya Municipal Hospital, 39-1 Asahigaokacho, Ashiya, Hyogo, 659-8502 Japan; 7 Department of Orthopaedic Surgery, Osaka National Hospital, 2-1-14 Hoenzaka, Chuo-ku, Osaka, 540-0006 Japan

Correction to: *Scientific Reports* 10.1038/s41598-019-52143-3, published online 01 November 2019

The original version of this Article contained an error throughout the Article, where the number “4” was incorrectly subscripted in “DUX4”. This has now been corrected in the PDF and HTML versions of the Article. The Supplementary Information file was correct at time of publication.

