# Supplementary material for: Establishment of a novel human CIC-DUX4 sarcoma cell line, Kitra-SRS, with autocrine IGF-1R activation and metastatic potential to the lungs
Source: Sci Rep. 2019 Nov 1;9:15812. doi: 10.1038/s41598-019-52143-3 (PMC6825133; doi:10.1038/s41598-019-52143-3)
Supplement: Supplementary file 1 — Supplementary information [file 41598_2019_52143_MOESM1_ESM.pdf]

Supplementary Information

Establishment of a novel human CIC-DUX4 sarcoma cell line, Kitra-SRS, with autocrine IGF-1R activation and metastatic potential to the lungs

Sho Nakai[1], Shutaro Yamada[2], Hidetatsu Outani[1], Takaaki Nakai[3], Naohiro Yasuda[1], Hirokazu Mae[1], Yoshinori Imura[4], Toru Wakamatsu[4], Hironari Tamiya[4], Takaaki Tanaka[4], Kenichiro Hamada[1], Akiyoshi Tani[5], Akira Myoui[1], Nobuhito Araki[6], Takafumi Ueda[7], Hideki Yoshikawa[1], Satoshi Takenaka[1]*, Norifumi Naka[1, 4]

[1] Department of Orthopaedic Surgery, Osaka University Graduate School of Medicine, 2-2 Yamadaoka, Suita, Osaka 565-0871, Japan

[2] Department of Orthopaedic Surgery, Yao Municipal Hospital, 1-3-1 Ryugecho, Yao, Osaka 581-0069, Japan

[3] Department of Orthopaedic Surgery, Kawachi General Hospital, 1-31 Yokomakura, Higashiosaka, Osaka 578-0954, Japan

[4] Musculoskeletal Oncology Service, Osaka International Cancer Institute, 3-1-69 Otemae, Chuo-ku, Osaka 541-8567, Japan

[5] Compound Library Screening Center, Osaka University Graduate School of Pharmaceutical Sciences, 1-6 Yamadaoka, Suita, Osaka 565-0871, Japan

[6] Department of Orthopaedic Surgery, Ashiya Municipal Hospital, 39-1 Asahigaokacho, Ashiya, Hyogo 659-8502, Japan

[7] Department of Orthopaedic Surgery, Osaka National Hospital, 2-1-14 Hoenzaka, Chuo-ku, Osaka 540-0006, Japan

*Corresponding author: Satoshi Takenaka

Department of Orthopaedic Surgery, Osaka University Graduate School of Medicine

2-2 Yamadaoka, Suita, Osaka 565-0871, Japan, Phone: +81-6-6879-3552; Fax: +81-6-6879-3559

E-mail: s.takenaka.0816@ort.med.osaka-u.ac.jp

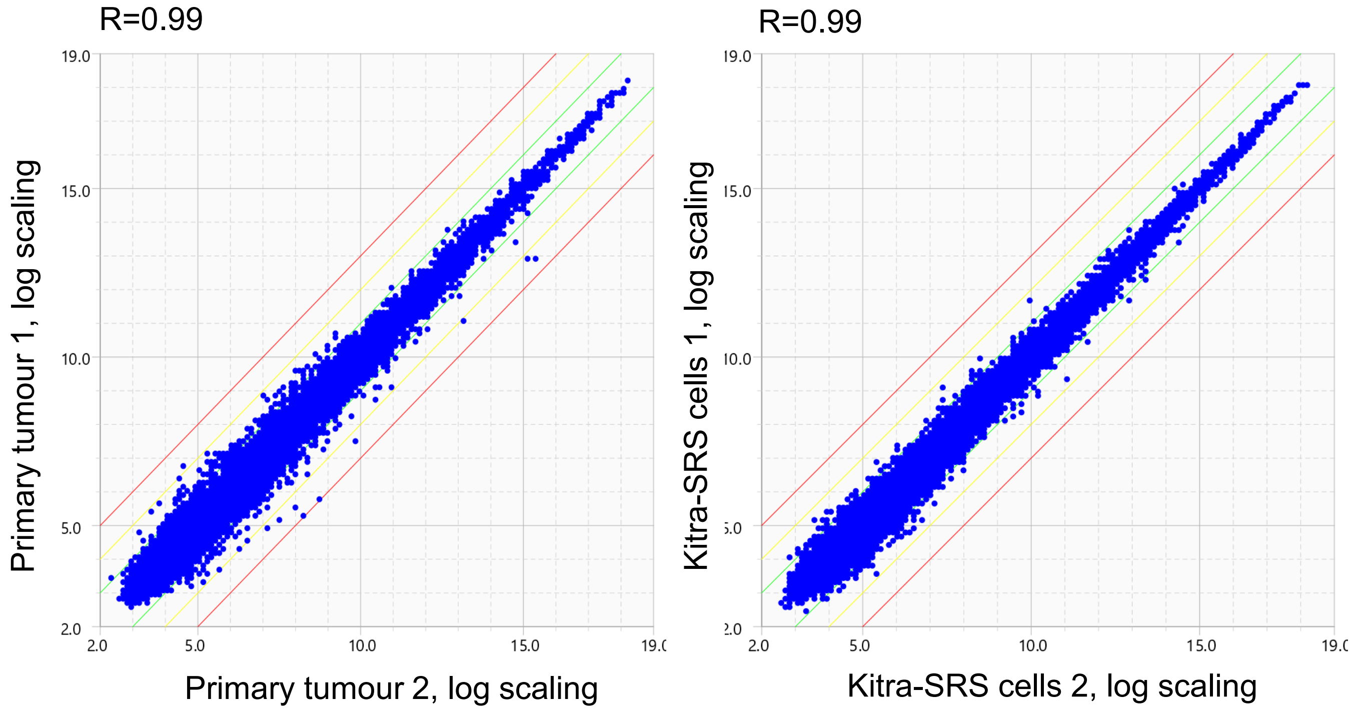

Supplementary Figure S1. Scatter plots showing the correlation of gene expression between samples of the primary tumour (left) and the correlation between samples of Kitra-SRS cells (right). R represents the correlation coefficient. Green and red lines indicate absolute log-fold-change >1 and >3, respectively.

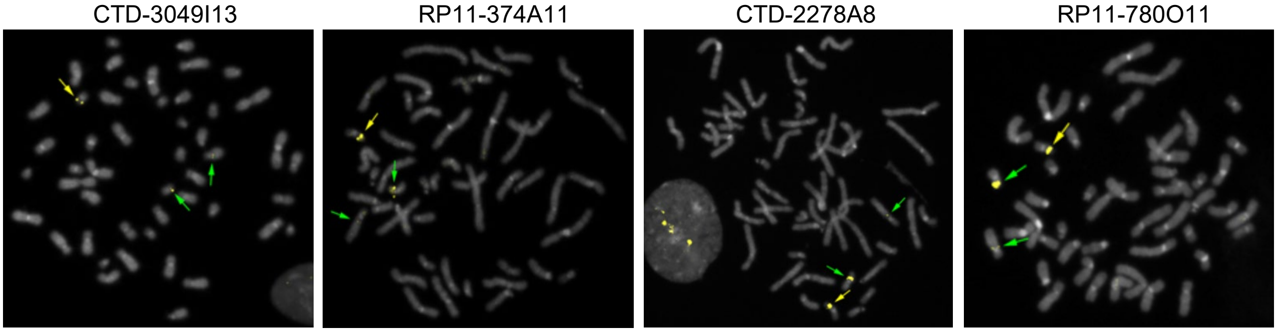

Supplementary Figure S2. FISH analysis of Kitra-SRS cells. The yellow arrows indicate probes for the 19q13.2 region; the green arrows, a split of one copy of each probe.

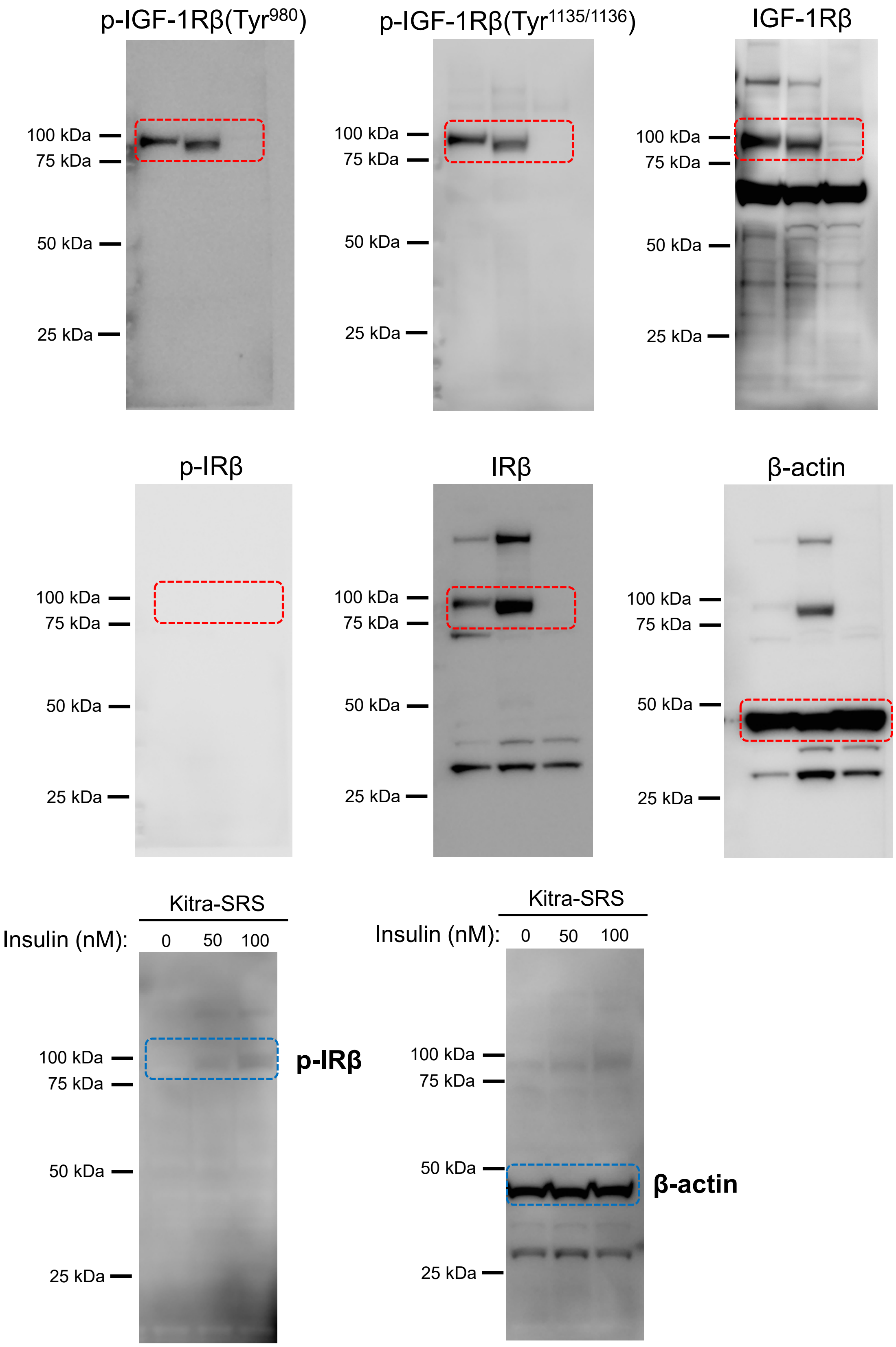

Supplementary Figure S3. Full-length blots of p-IGF-1Rβ(Tyr^{980}), p-IGF-1Rβ(Tyr$^{1135/1136}$), IGF-1Rβ, p-IRβ, IRβ and β-actin protein expression for Figure 5b. Kitra-SRS cells were treated with 0-100 nM insulin for 10 min and then subjected to western blotting with the anti-p-IRβ antibody. Insulin was used as a positive control.

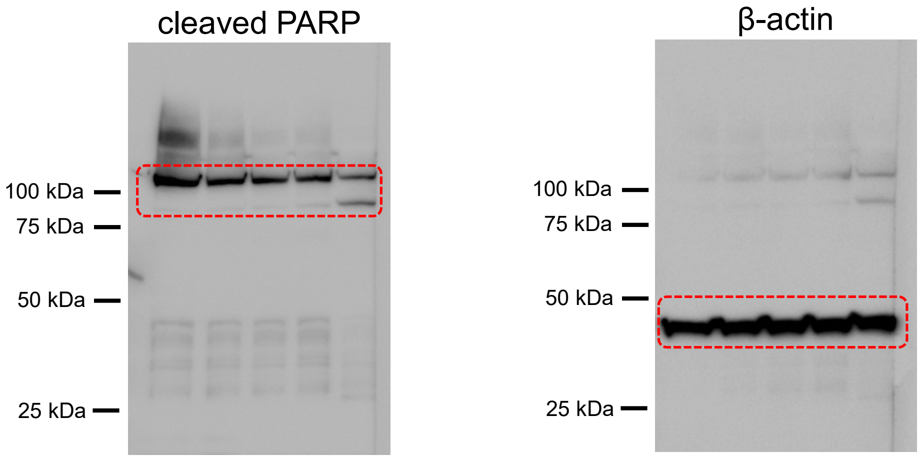

Supplementary Figure S4. Full-length blots of cleaved PARP and β-actin protein expression for Figure 6c.

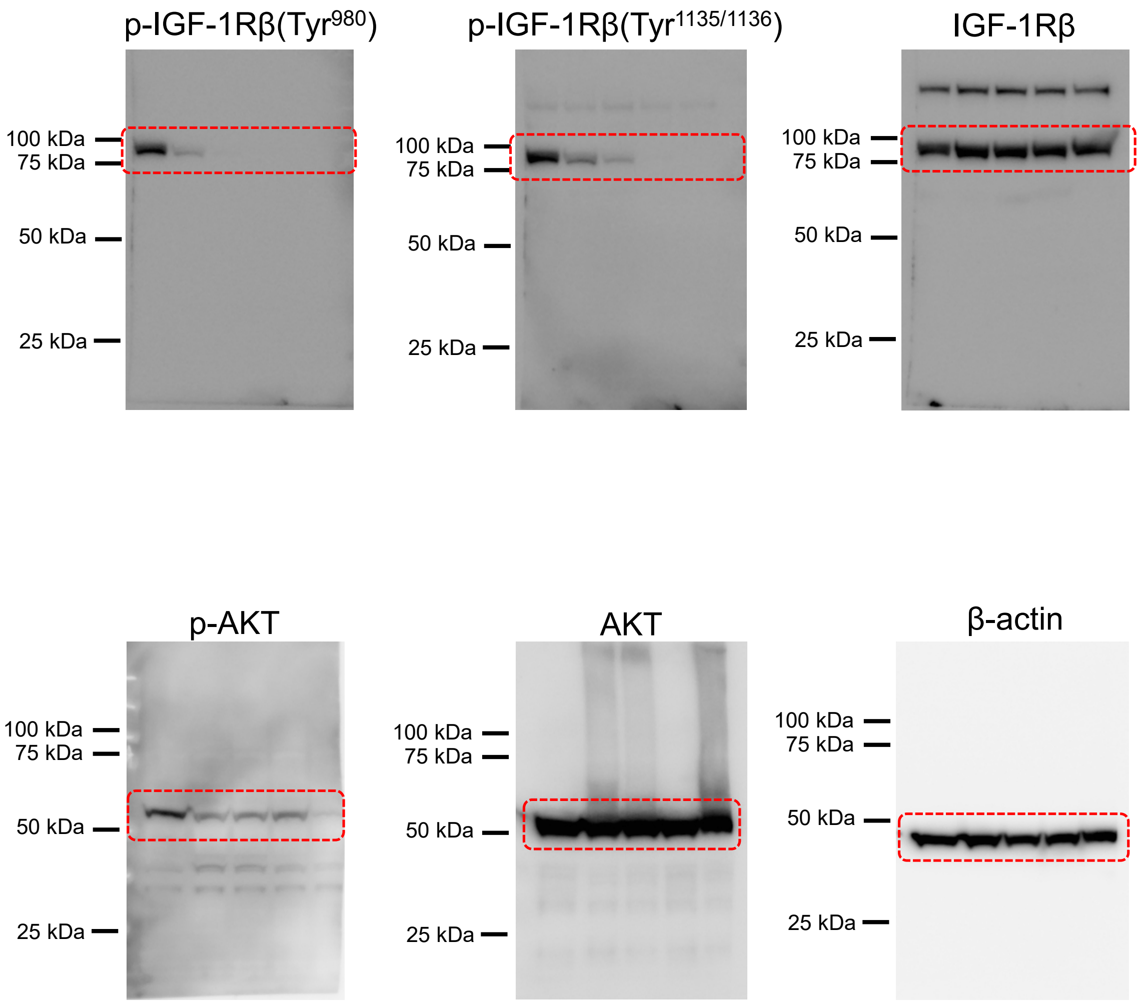

Supplementary Figure S5. Full-length blots of p-IGF-1Rβ(Tyr^{980}), p-IGF-1Rβ($Tyr^{1135/1136}$), IGF-1Rβ, p-AKT, AKT and β-actin protein expression for Figure 6d.

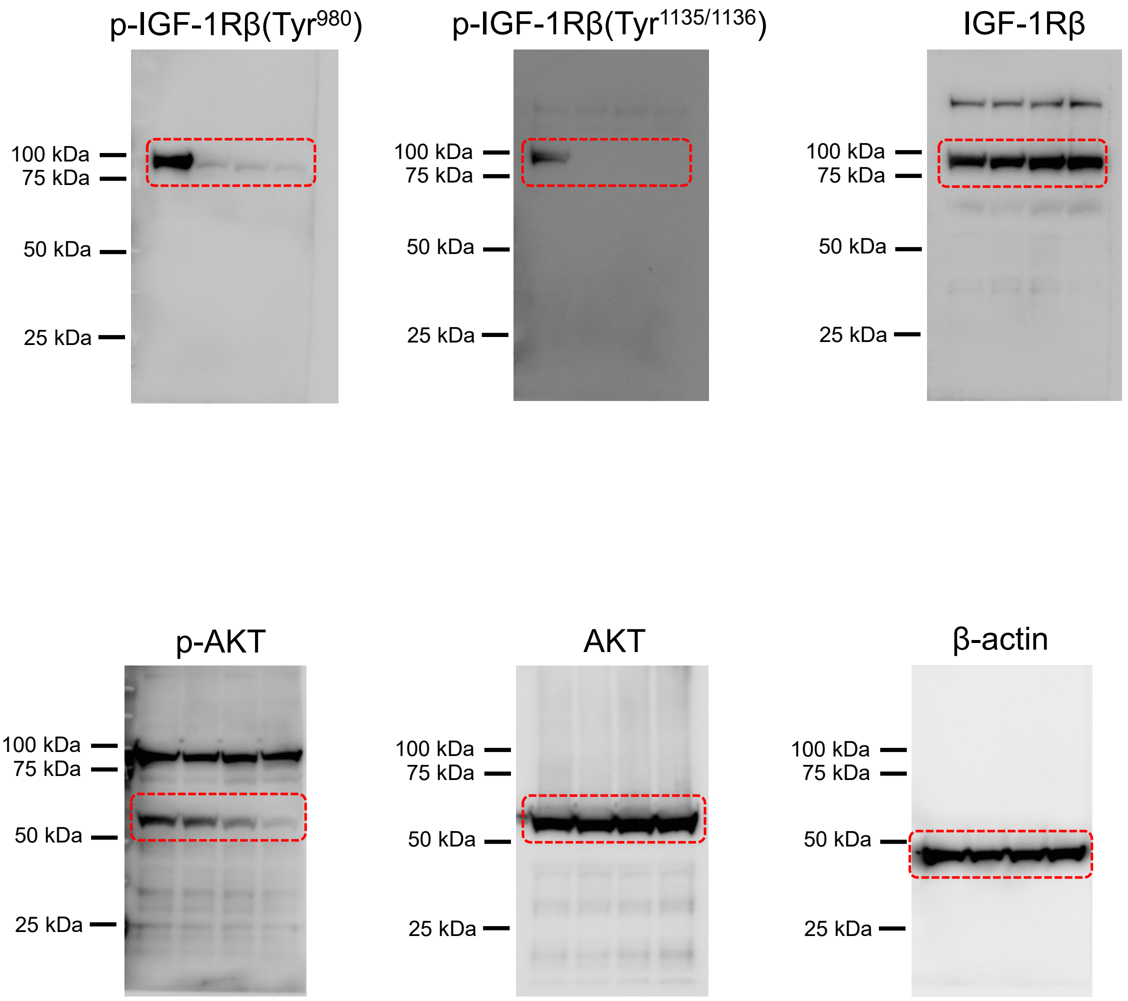

Supplementary Figure S6. Full-length blots of p-IGF-1Rβ(Tyr^{980}), p-IGF-1Rβ(Tyr$^{1135/1136}$), IGF-1Rβ, p-AKT, AKT and β-actin protein expression for Figure 6e.

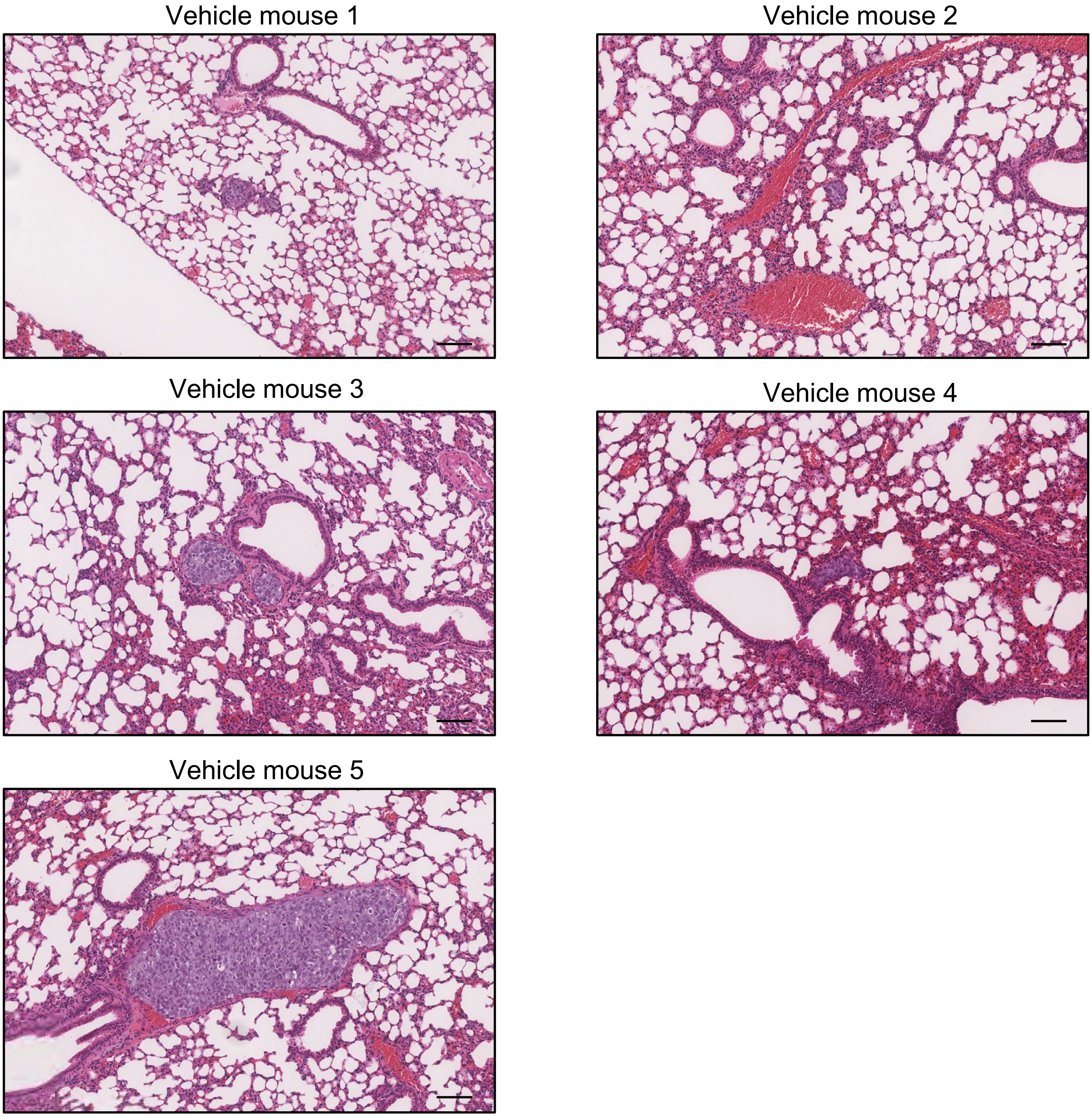

Supplementary figure S7. HE staining of lungs in vehicle-treated mice. Scale bars: 100μm.

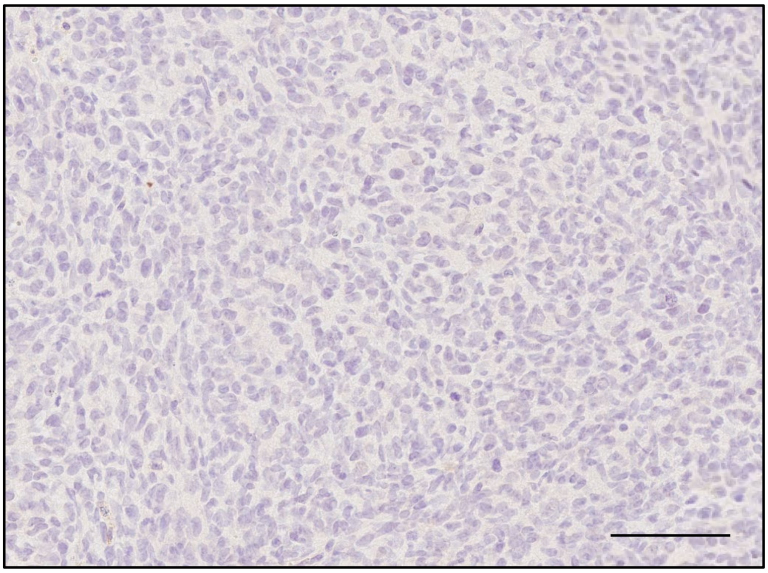

Supplementary Figure S8. A negative control for Ki-67 immunostaining.

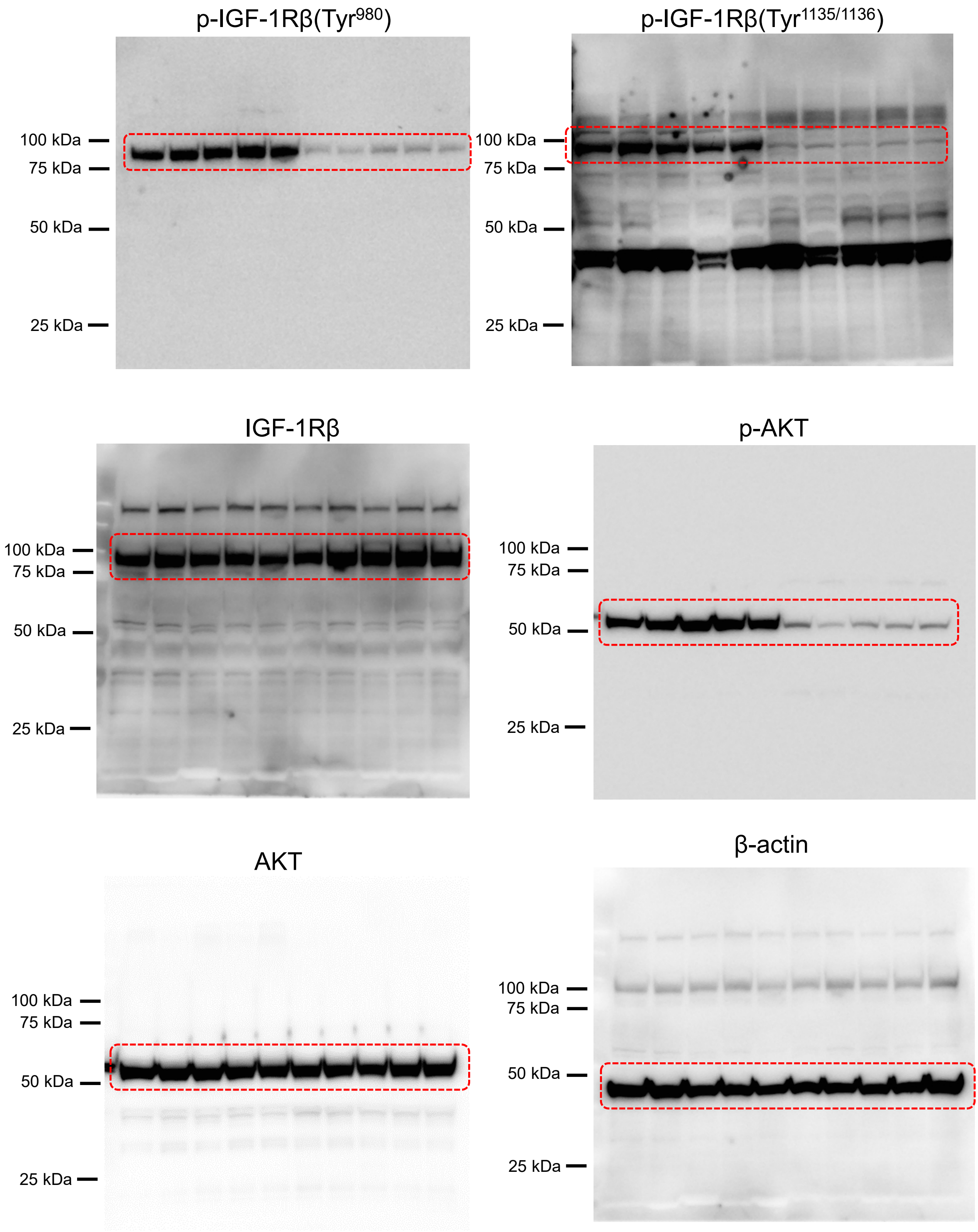

Supplementary Figure S9. Full-length blots of p-IGF-1Rβ(Tyr^{980}), p-IGF-1Rβ(Tyr$^{1135/1136}$), IGF-1Rβ, p-AKT, AKT and β-actin protein expression for Figure 7g.

Supplementary Table S1. STR analysis.

Microsatellite (Chromosome)	Kitra-SRS (P50)	Tumor Tissue
Amelogenin	X, X	X, X
D21S11	29, 32.2	29, 32.2
D5S818	10, 12	10, 12
D13S317	8, 10	8, 10
D7S820	11, 12	11, 12
D16S539	9	9
vWA	16, 18	16, 18
TH01	7, 9	7, 9
TPOX	8, 9	8, 9
CSF1PO	12	12

Cells were compared to a tumor tissue from the patient.

P indicates a passage number of a cell line.

Supplementary Table S2. Detection of *CIC-DUX4* from RNA-seq data.

Kitra-SRS	*CIC*(4525)-*DUX4L2*(1075)<br>*CIC*(4376)-*DUX4L4*(1014)<br>*CIC*(4505)-*DUX4L12*(977)

Numbers in parentheses represent the nucleotide position at the breakpoint junction in the reference cDNA sequence of each gene; *CIC* (NM_015125), *DUX4L2* (NM_001127386), *DUX4L4* (NM_001177376), and *DUX4L12* (NG_012776).

Supplementary Table S3. Primer sequences for the RT-PCR.

Primer name	Primer sequence (5’->3’)
**CIC4120(ref)**	TGAGTTGCCTGAGTTTCG
**DUX4RTr2**	TGAGGGGTGCTTCCAGCG
**CIC-fl**	ACCATGTATTCGGCCCACAGG
**DUX4-fl**	TCCTAAAGCTCCTCCAGCAGAG
**pENTER1A-F**	CTACAAACTCTTCCTGTTAGTTAG
**pENTER1A-R**	ATGGCTCATAACACCCCTTG
**CIC770F**	AAGGAGAAGCAGAAGTACCACGAC
**CIC1522F**	GTTTTCACCTGTGATCCGTTCCTC
**CIC2309F**	CCTGCCACTGTCACTAACCTACTG
**CIC3046F**	CAGAATCACCTATGTGCAGTCAGC
**CIC3838F**	CAGCAAATTCCCCAGCTCATCTTC
**CIC4523F**	GCCCGCTATGCAGACATCTTTC

Supplementary Table S4. *DUX4* pseudogenes identical to the *DUX4* component of *CIC-DUX4* transcript of Kitra-SRS cells from Ensemble Genome Browser 95: http://www.ensembl.org/index.html.

Subject name	Gene	Genomic Location
ENST00000611059.1	*DUX4L15*	10:133760834-133761125
ENST00000554103.2	*DUX4L13*	10:133754225-133754516
ENST00000622460.1	*DUX4L10*	10:133744307-133744598
ENST00000619712.1	*DUX4L20*	10:133684645-133684936
ENST00000622058.1	*DUX4L21*	10:133681346-133681637
ENST00000618238.1	*DUX4L22*	10:133678036-133678327
ENST00000615195.1	*DUX4L23*	10:133674726-133675017
ENST00000617576.2	*DUX4L24*	10:133671427-133671718
ENST00000624915.1	*DUX4L25*	10:133668128-133668419
ENST00000566884.2	*DUX4L4*	4:190082095-190082386

Supplementary Table S5. Karyotype results of 10 metaphase cells on Kitra-SRS cells at passage 20 in M-FISH analysis.

Karyotype results	Cell number
48, XX, del(1)(p32), +8, t(12;19)(q13;q13), +20	6
48, XX, +8, t(12;19)(q13;q13), +20	2
47, XX, del(1)(p32), +8, t(12;19)(q13;q13), -15, +20	1
47, XX, del(1)(p32), t(12;19)(q13;q13), +20	1

Supplementary Table S6. Karyotype results of 20 metaphase cells on Kitra-SRS cells at passage 100 in G-banding.

Karyotype results	Cell number
47,XX,del(1)(p?),+8,der(12)add(12)(p13)t(12;19)(q13;q13.1), der(19)t(12;19)(q13;q13.1)	15
49,XX,+1,del(1)(p?)x2,+8,t(12;19)(q13;q13.1),+20	1
48,XX,del(1)(p?),+8,t(12;19)(q13;q13.1),+20	1
48,XX,del(1)(p?),+8,+der(12)t(12;19)(q13;q13.1), t(12;19)(q13;q13.1),+20	1
48,XX,del(1),+3,+8,der(12)add(12)t(12;19)(q13;q13.1), add(17)(q11.2),der(19)t(12;19) (q13;q13.1)	1
49,XX,del(1),+8,der(12)add(12)t(12;19)(q13;q13.1), +?19,der(19)t(12;19),+20	1

Supplementary Table S7. Incidence of lung metastasis in Kitra-SRS-inoculated mice.

	Metastasis +
1×10^{7} cells (n=5)	4/5 (80%)
1×10^{8} cells (n=5)	3/5 (60%)

Supplementary Table S8. Seventeen drugs that inhibited more than 80 % cell viability in Kitra-SRS cells among 1134 FDA-approved drugs.

Drug name	Cell viability (%)
	Kitra
Mitoxantrone HCl	-3.2
Penfluridol	0.1
Alexidine HCl	0.5
Auranofin	0.5
Terfenadine	1.2
Ponatinib (AP24534)	2
Carfilzomib (PR-171)	2.3
Emetine	2.3
Idarubicin HCl	3.2
Daunorubicin HCl	3.5
Bortezomib (PS-341)	3.6
9-Aminoacridine	3.9
Crystal Violet	4.2
Doxorubicin (Adriamycin)	6.6
Epirubicin HCl	7
Topotecan HCl	7.8
Camptothecin	8.3

Supplementary Table S9. Antibodies.

Target	Clone/ product name	Source	Supplier	Concentration/ Dilution	Blocking buffer
Immunohistochemistry					
CD99	O13	mouse monoclonal	Nichirei Biosciences	a diluted antibody	-
bcl-2	124	mouse monoclonal	Nichirei Biosciences	a diluted antibody	-
WT1	#83535	rabbit monoclonal	Cell Signaling Technology	1:100	-
Ki-67	#9027	rabbit monoclonal	Cell Signaling Technology	1:400	-
normal rabbit IgG	148-09551	-	Wako	1:1000	-
normal mouse IgG	sc-2025	-	Santa Cruz	1:1000	-
Immunoblotting					
IGF-1Rβ	#3027	rabbit monoclonal	Cell Signaling Technology	1:1000	5% milk in TBST
phospho-IGF-1Rβ (Tyr980)	#4568	rabbit monoclonal	Cell Signaling Technology	1:1000	5% milk in TBST
phospho-IGF-1Rβ (Tyr1135/1136)	#3024	rabbit monoclonal	Cell Signaling Technology	1:1000	5% milk in TBST
IRβ	#3025	rabbit monoclonal	Cell Signaling Technology	1:1000	5% milk in TBST
phospho-IRβ (Tyr1185)	ab62321	rabbit monoclonal	Abcam	1:2000	5% milk in TBST
AKT	#4691	rabbit monoclonal	Cell Signaling Technology	1:1000	5% milk in TBST
phospho-AKT	#4060	rabbit monoclonal	Cell Signaling Technology	1:1000	5% milk in TBST
cleaved PARP	#9542	rabbit monoclonal	Cell Signaling Technology	1:1000	5% milk in TBST
β-actin	#4970	rabbit monoclonal	Cell Signaling Technology	1:1000	5% milk in TBST
horseradish peroxidase (HRP)-coupled goat anti-rabbit IgG	#7074	-	Cell Signaling Technology	1:1000	-
HRP-coupled horse anti-mouse IgG	#7076	-	Cell Signaling Technology	1:1000	-
